# CLARISA: Connexin-43 Lateralization Automated ROI-Based Image Signal Analyzer

**DOI:** 10.3390/ijms27115033

**Published:** 2026-06-02

**Authors:** Daniel Gattari, Joseba Sancho-Zamora, Debora Chan, Natalia Jorgelina Prado, Emiliano Raúl Diez, Mariano Llamedo Soria, Mario Rossi

**Affiliations:** 1 Faculty of Engineering, Austral University, Pilar B1629WWA, Buenos Aires, Argentina; dgattari@austral.edu.ar (D.G.); dchan@austral.edu.ar (D.C.); 2 Functional Genomics and Data Science, Institute for Translational Medicine Research (IIMT), CONICET-Universidad Austral, Pilar B1629AHJ, Buenos Aires, Argentina; 3 Tecnun School of Engineering, Universidad de Navarra, 31009 Donostia, Spain; jsanchoz@unav.es; 4 Institute of Experimental Medicine and Biology of Cuyo (IMBECU), CONICET, Facultad de Ciencias Médicas, Universidad Nacional de Cuyo, Mendoza 5500, Argentina; natalia.prado@fcm.uncu.edu.ar (N.J.P.); diez.emiliano@fcm.uncu.edu.ar (E.R.D.); 5 Electronics Department, National Technological University, Buenos Aires C1041AAJ, Argentina; llamedom@frba.utn.edu.ar

**Keywords:** connexin-43, lateralization, deep learning, multi-scale classification, fluorescence microscopy, automated quantification

## Abstract

Connexin-43 (CX43) lateralization in ventricular myocardium has been associated with abnormal impulse propagation and increased arrhythmia susceptibility. Its quantitative assessment in histological sections remains challenging because previous methods require segmentation of individual cardiomyocytes and rely on geometric rules applied to segmented cell profiles. Here, we present CLARISA, a segmentation-free, ROI-based deep learning framework that classifies CX43-positive regions as terminal or lateralized directly from fluorescence images. An expert-annotated dataset was generated from left-ventricular cryosections of Wistar rat hearts, in which CX43-positive regions were labeled according to their distribution pattern. A dual-stream EfficientNetV2-S classifier was trained to capture both local and contextual ROI morphology. We also developed a semi-automated whole-section inference module to generate spatial lateralization probability maps and global percent lateralization estimates. On the held-out test set, CLARISA achieved a ROC-AUC of 0.904 (95% bootstrap CI: 0.828–0.960) and a PR-AUC of 0.808 (95% bootstrap CI: 0.682–0.913), supporting the feasibility of automated ROI classification for CX43 lateralization assessment. When deployed on whole tissue sections, including an independently analyzed section not used during model development, CLARISA generated spatial maps that captured heterogeneous CX43 organization and produced a global percent lateralization estimate closely aligned with expert annotation, differing by only 1.30 percentage points over the same detected CX43-positive area. Comparison with a previously published segmentation-based method further indicated that ROI-based and cell-segmentation-based approaches provide related but non-equivalent readouts of CX43 lateralization. The ROI-based design additionally reduces annotation burden—requiring classification of discrete CX43-positive signal rather than complex cardiomyocyte delineation—and ensures that all detected CX43-positive signal contributes to the lateralization estimate regardless of cell boundaries. These results establish CLARISA as a proof-of-principle framework for scalable, segmentation-free CX43 lateralization assessment in cardiac tissue. Further validation across larger, independent, and more heterogeneous datasets will be required to assess robustness, portability across imaging conditions, and translational applicability. The complete codebase, pretrained model, image data, and expert annotation tool are publicly available.

## 1. Introduction

Cardiac impulse propagation depends on multiple structural and functional determinants. Among these, cardiomyocyte size and shape play a central role [1,2]. At the cellular level, membrane area contributes to capacitive properties, whereas cell volume influences intra-cellular resistance. Beyond the single cell, conduction is further modulated by tissue-level organization, including the spatial arrangement of myocytes, non-myocyte cells, and connective tissue within the extracellular space [3]. At the molecular level, impulse propagation is critically shaped by the expression and spatial distribution of connexins.

Connexins form intercellular channels at gap junctions, providing direct electrical and metabolic coupling between adjacent cardiomyocytes and thereby sustaining the syncytial behavior of the myocardium [4]. In healthy ventricular tissue, connexin-43 (CX43) is predominantly concentrated at the intercalated discs located at the longitudinal ends of cardiomyocytes. Under pathological or stress conditions, however, CX43 may redistribute toward the lateral cell borders, a phenomenon commonly referred to as lateralization [5].

Because the spatial arrangement of gap junctions directly influences both the strength and anisotropy of intercellular coupling, changes in CX43 distribution are considered functionally relevant. In diseased myocardium, increased lateralization, often accompanied by an overall reduction in CX43 expression, has been associated with abnormal impulse propagation and increased susceptibility to arrhythmias [6]. However, the functional consequences of this redistribution remain debated, as some studies suggest that laterally displaced CX43 may partially compensate for structural inhomogeneities by preserving some degree of coupling in remodeled regions [7]. This unresolved issue highlights the need for quantitative approaches capable of characterizing CX43 distribution across large tissue areas.

Quantitative assessment of CX43 distribution in histological sections poses substantial practical challenges. The number of intercellular junctions within the myocardium far exceeds what can be comprehensively evaluated by conventional microscopy, while manual annotation is labor-intensive and therefore usually restricts analysis to small regions of interest. As a result, many studies may fail to capture the spatial heterogeneity of connexin remodeling across an entire tissue section [8]. A further challenge lies in the segmentation of individual cardiomyocytes in intact tissue. Their complex branching morphology, dense packing, and irregular boundaries make reliable delineation difficult even for trained observers [9]. In standard histological preparations, oblique sectioning and limited membrane contrast further increase the uncertainty associated with identifying individual cellular profiles [10].

These limitations have motivated the development of automated and semi-automated image analysis methods. In cardiac tissue, deep learning approaches have been applied to histopathological classification and cellular quantification [11,12,13]. More specialized methods have also been proposed to assess gap junction distribution in rat ventricular myocardium [14] and to evaluate CX43 localization through co-localization with N-cadherin in atrial tissue [15]. Automated quantification of intercellular coupling has likewise been reported in both non-cardiac cell cultures [16] and cardiac co-culture systems [17]. Despite these advances, no currently available method provides an automated framework for classifying CX43 distribution patterns across large myocardial tissue sections, particularly for distinguishing terminal from lateralized signal.

A method specifically designed for the automated quantification of CX43 lateralization in fluorescence immunohistochemistry images of ventricular myocardium has previously been reported [18]. For clarity, this method is hereafter referred to as MARTA. In that approach, explicit segmentation of individual cardiomyocytes was required as a prerequisite for quantification. Once cell contours had been identified, each cell area was subdivided into terminal and central compartments using geometric rules, and the CX43 signal was assigned accordingly to estimate a lateral-to-total ratio. This design imposed several limitations. First, quantification was restricted to regions in which cardiomyocytes could be successfully segmented, leaving any CX43 signal outside delineated cells unassigned. Second, the method relied on a fixed geometric partition of the cell profile, assuming that compartment boundaries derived from a bounding rectangle adequately represented the underlying cellular morphology. Third, the pipeline consisted of a predefined sequence of morphological operations, including dilation, erosion, and contour extraction, and therefore did not learn from data, potentially limiting its robustness across images with different staining characteristics or tissue properties. Finally, extending this framework to supervised learning would require instance-level segmentation masks of individual cardiomyocytes, a type of annotation that is particularly difficult to generate in intact myocardial tissue.

Taken together, these considerations support the development of complementary strategies that reduce dependence on cardiomyocyte segmentation, without implying that segmentation-based methods are unsuitable in all contexts.

In the present work, we introduce CLARISA (Connexin Lateralization Automated ROI-based Image Signal Analyzer), an ROI-based approach in which CX43-positive regions, rather than cardiomyocytes, are the primary unit of analysis. Each region is classified directly as terminal or lateralized based on its appearance within the surrounding tissue context. This reframing also simplifies annotation: experts only need to identify CX43-positive regions and assign each to one of two classes—a substantially lighter task than producing cell-level segmentation masks, since CX43-positive regions appear as discrete, well-delimited structures, whereas cardiomyocyte boundaries are often ambiguous in intact tissue. This lighter annotation burden makes it feasible to build a labeled dataset, which in turn enables training a deep learning classifier that learns the morphological features distinguishing lateralized from terminal signal. At inference, the classifier assigns a lateralization probability to each detected CX43-positive region, independently of whether it falls within a delineated cell.

**Figure 1:**
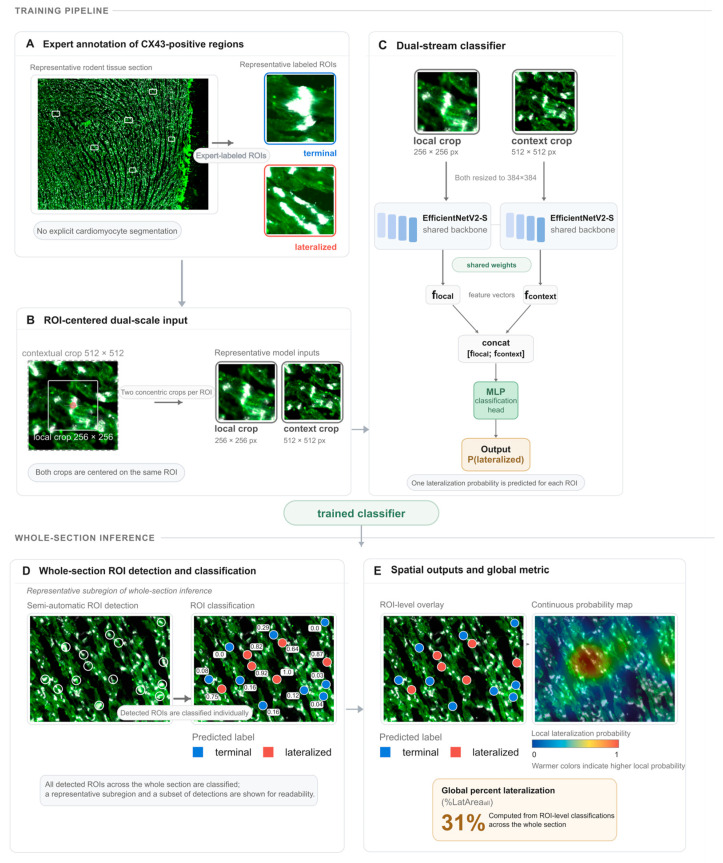
Overview of the proposed methodological framework, CLARISA, for the quantification of CX43 lateralization in histological images of cardiac tissue. The workflow is organized in two stages: a training pipeline (**top**) in which a classifier is learned from expert-annotated data, and a whole-section inference pipeline (**bottom**) in which the trained classifier is applied to previously unseen tissue sections. (**A**) Expert annotation of CX43-positive regions. On representative rodent ventricular tissue sections, the expert manually selected regions of interest (ROIs) around CX43-positive signal and assigned each of them to one of two classes: terminal, when the signal was located at the longitudinal ends of the cardiomyocyte, or lateralized, when it was located along its lateral membrane. Annotation was performed directly on the fluorescence image, without explicit segmentation of cardiomyocyte contours. (**B**) ROI-centered dual-scale input. For each annotated ROI, two spatially aligned square crops centered on the same location were extracted from the tissue section: a local crop capturing the CX43-positive region itself and its immediate surroundings, and a context crop capturing a larger portion of surrounding tissue. (**C**) Dual-stream classifier. The local and context crops, both resized to 384 × 384 px, were encoded by an EfficientNetV2-S backbone with shared weights across the two streams, yielding two feature vectors that were concatenated and passed to a multi-layer perceptron (MLP) classification head. The classifier output was a single scalar, P(lateralized), representing the predicted probability that the input ROI belongs to the lateralized class. (**D**) Whole-section ROI detection and classification. When applied to a new tissue section, the trained classifier is embedded in a semi-automated inference workflow that first detects candidate CX43-positive regions using adjustable image-processing parameters and then classifies each detected ROI individually, producing a predicted probability and a final class label per region. The panel illustrates this step on a representative subregion of the whole section, with per-ROI probabilities shown on a subset of detections for readability. (**E**) Spatial outputs and global metric. The per-ROI classifications are aggregated into two complementary representations of the predicted lateralization pattern across the tissue section—a discrete ROI-level overlay coloring each detected region by its assigned class, and a continuous probability map obtained by Gaussian spatial interpolation of the per-ROI probabilities (warmer colors indicate higher local lateralization probability)—and summarized by a single global metric, %LatArea_all_, defined as the fraction of the total detected CX43-positive area assigned to the lateralized class. The illustrative value shown in the panel corresponds to the representative subregion and does not reflect any specific experimental result reported in this work.

CLARISA’s framework comprises three main components: (i) generation of an expert-annotated dataset of CX43-positive regions labeled as terminal or lateralized; (ii) training of a deep learning classifier, based on a pretrained backbone, to predict the lateralization probability of individual regions from their local and contextual image appearance; and (iii) a semi-automated whole-section inference module that detects candidate regions using adjustable image-processing parameters and classifies them with the trained model, yielding an estimate of the lateralized fraction within the detected CX43-positive area. In addition, the framework includes an interactive annotation tool intended to facilitate the creation and extension of ROI-level datasets in future applications. Spatial probability maps are also generated to support visual inspection of predicted lateralization patterns. The approach does not require explicit cardiomyocyte segmentation and is presented here as a proof-of-principle framework rather than as a fully automated or broadly generalizable tool. An overview is provided in Figure 1.

The aim of this study was to develop and evaluate CLARISA as a segmentation-free, ROI-based methodological framework for CX43 lateralization assessment in fluorescence immunohistochemistry images of rodent ventricular myocardium. The classifier was evaluated at the level of individual CX43-positive regions using held-out tissue sections, and whole-section applicability was assessed through the semi-automated inference module described above, including an exploratory comparison with the previously developed MARTA framework. The method, together with the annotated dataset, expert annotation tool, pretrained model, and code required to reproduce the pipeline, has been made publicly available through the resources listed in the Supplementary Materials. Together, these resources are intended to provide a reproducible baseline that can be inspected, extended, or retrained with additional annotations for future datasets acquired under different experimental conditions.

## 2. Results

### 2.1. Architecture and Input Representation Optimization

The classifier was trained to discriminate between terminal and lateralized CX43-positive regions from a dual-scale image representation: a local crop centered on each annotated region and a contextual crop capturing the surrounding tissue (Section 4.3). Feature extraction was performed with a shared EfficientNetV2-S backbone; the resulting feature vectors for both input scales were concatenated and processed by a multi-layer perceptron (MLP) classification head (Section 4.5.2). Architecture and training hyperparameters were optimized with Optuna in two sequential stages—an architecture search followed by a fine-tuning search—using a validation set of 68 ROIs from tissue section IM1315, withheld from all training (Section 4.5.4).

#### 2.1.1. Architecture Search Identified the Importance of Tissue Context

The first optimization stage showed that local-only models consistently underperformed architectures incorporating broader tissue context, indicating that the appearance of the CX43-positive focus alone was insufficient for robust discrimination between terminal and lateralized patterns. By contrast, both contextual-only and dual-scale formulations achieved better validation performance. The lowest validation loss in this stage was obtained by a contextual-only MLP model (validation loss = 0.0451), whereas the highest validation AUC was achieved by a dual-scale MLP model (validation AUC = 0.8389), which also retained similarly low validation loss. In addition, MLP-based heads consistently outperformed logistic heads among the top-ranked configurations, supporting the use of a non-linear decision layer. Taken together, these results indicate that successful classification benefits from both local morphology and tissue context. Accordingly, the dual-scale MLP architecture was selected as the base model for subsequent fine-tuning, as it combined competitive validation loss with the highest discriminative performance. Full trial-wise results are provided in Supplementary File S1 (architecture).

#### 2.1.2. Fine-Tuning Optimization Further Improved Validation Performance

After fixing the base architecture, a second Optuna study was performed to optimize training-related hyperparameters. This stage further improved validation performance, reducing the best validation loss by approximately 10.4% and increasing validation AUC from 0.832 to 0.862. The top-performing configurations consistently favored stronger regularization and a longer final fine-tuning stage, suggesting that performance benefited from extended joint adaptation of the full network after the earlier staged specialization steps. These configurations also supported the use of discriminative learning rates, with larger effective updates in the classification head and more conservative updates in the pretrained backbone. In addition, the best checkpoints were typically reached during stage 3, when the full network was unfrozen. Together, these results indicate that, beyond architectural design, classifier performance depended on progressively adapting the pretrained backbone to the morphological characteristics of CX43-positive regions. Full trial-wise results are provided in Supplementary File S1 (fine-tuning).

### 2.2. Final Model Performance on Held-Out Slides

**Figure 2:**
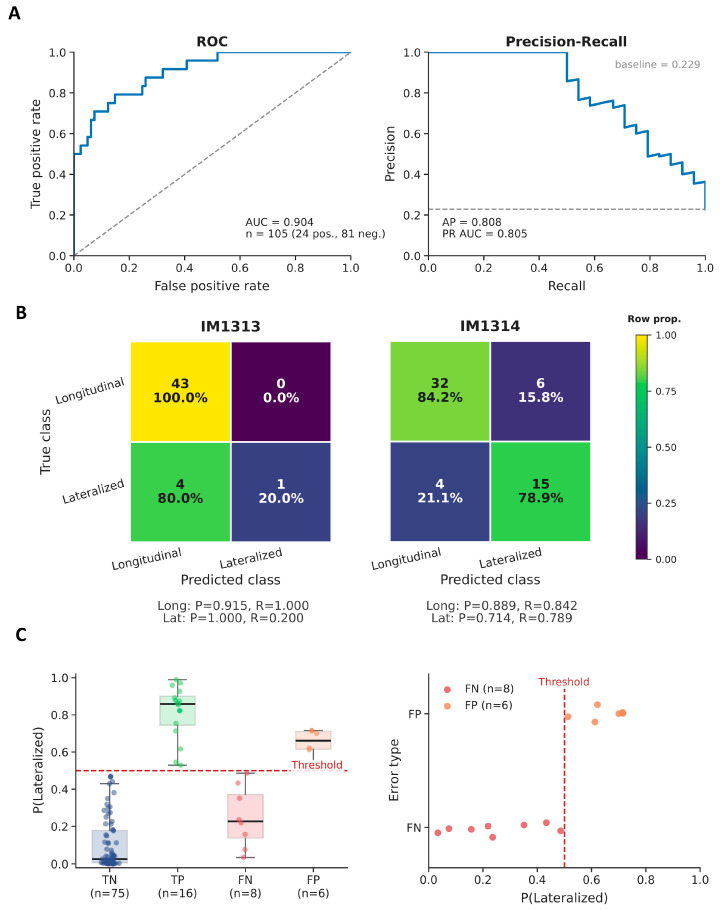
Quantitative performance of the final CLARISA classifier on the held-out test sections. (**A**) Global discrimination performance on the combined test set, shown as ROC and precision–recall curves. (**B**) Slide-level confusion matrices for the held-out test slides IM1313 and IM1314. Rows correspond to the true class and columns to the predicted class. Cell colors indicate row-normalized proportions, allowing direct comparison of classification behavior across slides despite differences in class distribution. Each cell reports both the absolute number of ROIs and the corresponding within-row percentage. Precision and recall for the terminal (longitudinal) and lateralized classes are reported below each panel. (**C**) ROI-level probability distributions in the test set. Left, distribution of predicted probabilities for the lateralized class grouped by prediction outcome (TN, TP, FN, FP). Right, predicted probabilities for false negatives and false positives relative to the default decision threshold of 0.5. Together, these panels show that correct predictions tend to occupy more extreme probability ranges, whereas misclassifications are enriched near the decision boundary.

The final selected model configuration was evaluated on the held-out test set, comprising 105 annotated ROIs from tissue sections IM1313 and IM1314, which were withheld from all stages of model development (Section 4.5.1). On the combined test set, the model achieved a ROC-AUC of 0.904 (95% bootstrap CI: 0.828–0.960) and a PR-AUC of 0.808 (95% bootstrap CI: 0.682–0.913; Figure 2A). These confidence intervals support the presence of a meaningful discriminative signal in the held-out test set, although they also highlight the uncertainty associated with performance estimation from a limited number of annotated ROIs. Overall, the model performed well on the terminal class, with somewhat lower performance on the lateralized class.

When performance was examined at the slide level, a more heterogeneous pattern emerged (see Figure 2B). In IM1313, the model showed a highly conservative behavior for the lateralized class: no false positives were observed, and terminal ROIs were recovered with perfect recall, but only one of five lateralized ROIs was correctly identified (Figure 2B—left). By contrast, IM1314 displayed a more balanced behavior, with 15 of 19 lateralized ROIs correctly classified, corresponding to a lateralized-class precision of 0.714 and recall of 0.789, although 6 false positives were also observed (Figure 2B—right). Thus, model performance was not homogeneous across the held-out test set but depended strongly on the slide being analyzed.

At the ROI level, the distribution of predicted probabilities further showed that correct predictions tended to occupy more extreme probability ranges, whereas misclassifications were enriched closer to the decision threshold (see Figure 2C—left). A similar slide-dependent pattern was also observed in the training and validation sets (Supplementary Figures S3–S5), suggesting that ROI-level classification difficulty is influenced not only by slide-specific morphological context, but also by the presence of visually ambiguous regions that may be challenging even at the annotation level. These cases are examined in more detail in the following Discussion section.

Given the limited number of ROIs available in the test set, the reported performance estimates and their confidence intervals should nevertheless be interpreted with appropriate caution. Additional performance details for the final retrained model on the training and validation sets are provided in Supplementary Section S2.1.

### 2.3. Annotation Consistency

An annotation consistency study was conducted to assess the stability of the expert labels underlying the development dataset, following the procedure described in Section Annotation Consistency Assessment. A stratified subset of 180 CX43-positive ROIs, drawn from all six annotated tissue sections, was re-labeled by the same expert under blinded conditions after a 12-month interval. The repeated annotation agreed with the original labels in 154 of 180 ROIs (percent agreement = 85.56%). Cohen’s kappa was 0.703, indicating substantial intra-observer agreement [19]. Class-specific agreement was 83.78% for the terminal class and 88.41% for the lateralized class. Slide-level results are reported in Supplementary Table S7.

The same ROI subset was independently labeled by a second expert under identical blinded conditions. Agreement with the original annotation was observed in 150 of 180 ROIs (percent agreement = 83.33%), with a Cohen’s kappa of 0.651, also corresponding to substantial agreement. Class-specific agreement was 84.68% for the terminal class and 81.16% for the lateralized class. Overall, these results support a substantial level of annotation consistency for the binary classification of CX43 distribution patterns used in this study, while also indicating that agreement was not fully homogeneous across tissue sections (see Supplementary Section S2.2).

### 2.4. Deployment of the CLARISA Inference Pipeline on Held-Out Test Sections

To assess the behavior of the complete inference workflow (described in Section 4.6) at the tissue-section scale, the trained classifier was applied to two whole sections from the held-out test set, IM1313 and IM1314. Unlike the ROI-level evaluation described above, which was restricted to expert-annotated regions, this analysis considered all CX43-positive regions detected semi-automatically across each section. It therefore represents a deployment-oriented scenario in which detection, classification, spatial reconstruction, and global quantification are performed consecutively on the full available CX43-positive signal. In total, 3715 regions were processed in IM1313 and 1233 in IM1314, substantially exceeding the number of manually annotated ROIs contributed by these sections to the held-out test set (see Section 4.5.1).

**Figure 3:**
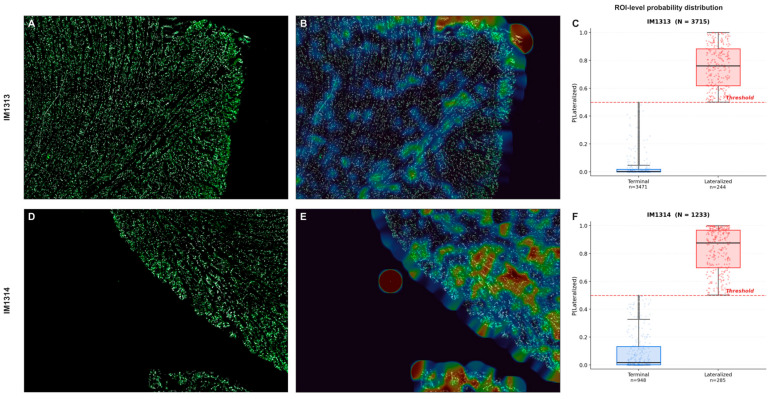
Whole-section inference outputs of the trained classifier on the two held-out test sections IM1313 (top row) and IM1314 (bottom row). (**A**,**D**) Original fluorescence images used as input for inference. (**B**,**E**) Continuous heatmaps of predicted lateralization generated from ROI-level probabilities by Gaussian kernel interpolation (k = 12, yielding σ ≈ 29 µm). Cooler colors indicate lower predicted lateralization, whereas warmer colors indicate higher predicted lateralization. (**C**,**F**) Boxplots of predicted lateralization probability (P(lateralized)) at the ROI level, partitioned by assigned class (terminal, P(lateralized) < 0.5; lateralized, P(lateralized) ≥ 0.5). Boxes show median and interquartile range; whiskers extend to 1.5× IQR; individual ROIs are shown as jittered points (subsampled for visualization when n > 300). The default decision threshold (0.5) is indicated by the dashed red line. The number of ROIs per class is reported below each box.

For each section, the inference module produced the complete set of outputs defined in Section 4.6.3 and Section 4.6.4: a continuous probability map of predicted lateralization, a discrete classification overlay assigning each detected ROI to either the terminal or lateralized class, and global section-level lateralization metrics. Figure 3 shows the original fluorescence images (A–D panels), the corresponding continuous lateralization heatmaps (panels B–E), and the ROI-level predicted probability distributions stratified by assigned class (panel C–F). In both sections, the heatmaps revealed spatially heterogeneous patterns of predicted CX43 lateralization rather than a uniform distribution across the tissue. This heterogeneity was more pronounced in IM1314, where localized regions of high predicted lateralization were clearly visible, whereas IM1313 showed a more homogeneous pattern with fewer and more scattered high-probability foci.

The ROI-level probability distributions further indicated that most classifications were made with probabilities clearly separated from the decision threshold (Figure 3C–F). In IM1313, ROIs assigned to the terminal class were strongly concentrated near zero probability values, with a median P(lateralized) of 0.001 and an interquartile range of 0.000–0.019. By contrast, ROIs assigned to the lateralized class showed substantially higher probabilities, with a median of 0.759 and an interquartile range of 0.618–0.883. A similar pattern was observed in IM1314, although with broader probability distributions: terminal ROIs had a median P(lateralized) of 0.017, with an interquartile range of 0.001–0.132, whereas lateralized ROIs had a median of 0.875, with an interquartile range of 0.698–0.968. Only a limited fraction of ROIs fell close to the decision boundary, defined here as P(lateralized) between 0.3 and 0.5: 3.15% in IM1313 and 8.27% in IM1314. Since inference was performed using the default binary decision threshold τ = 0.5, all detected ROIs were assigned to one of the two classes, and the *all* and *confident* variants of the global metrics were therefore identical for both sections.

The resulting whole-section metrics revealed marked differences between the two held-out sections. In IM1313, 3471 ROIs were classified as terminal and 244 as lateralized, corresponding to %LatArea_all_ = 6.63%. In IM1314, 948 ROIs were classified as terminal and 285 as lateralized, corresponding to %LatArea_all_ = 19.06%. Thus, despite being processed with the same inference workflow, IM1314 showed an approximately three-fold higher proportion of predicted lateralized CX43-positive area than IM1313 (see Table 1). These results illustrate that the proposed pipeline can be applied to complete tissue sections and can capture both local spatial heterogeneity and global differences in predicted CX43 lateralization across held-out samples.

### 2.5. Comparative Whole-Section Evaluation of CLARISA Against Expert Annotations and MARTA

We next evaluated the whole-section inference pipeline in a deployment setting beyond the images used for model development. For this purpose, we analyzed IM15, an independent tissue section acquired from a different global image and at a slightly different spatial resolution (0.3 μm/pixel; Section 4.1). CLARISA outputs were compared with manual annotations from a domain expert and with the previously published segmentation-based methodology, MARTA.

#### 2.5.1. CLARISA–Expert Annotation at ROI and Whole-Section Levels

The semi-automated ROI detection step used by CLARISA identified 359 CX43-positive regions in IM15. At the default decision threshold, τ = 0.5, all detected ROIs received a definitive CLARISA label, with no regions assigned to the indeterminate class. The expert independently labeled 185 of these ROIs as lateralized, 160 as terminal, and 14 as uncertain.

**Figure 4:**
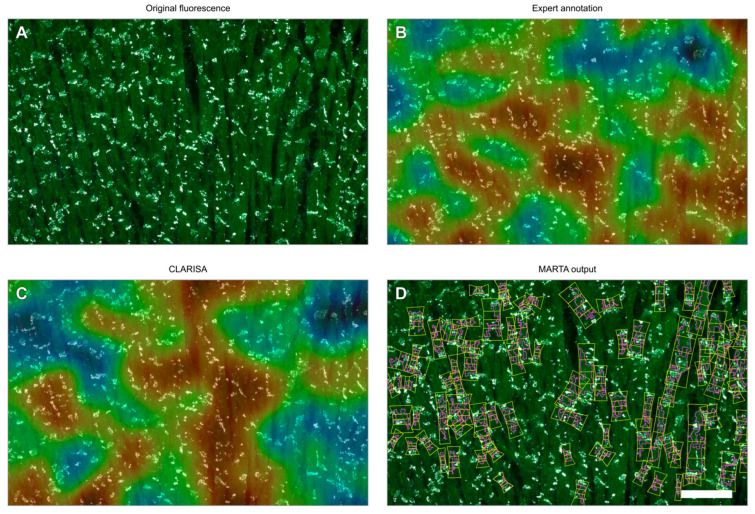
Whole-section comparison of CLARISA, expert annotation, and the MARTA segmentation-based methodology in IM15. (**A**) Original fluorescence image of the analyzed tissue section, used as the common reference for all visual outputs. (**B**) Continuous lateralization heatmap derived from expert annotations. Binary expert labels were converted into lateralization probabilities before interpolation, with terminal ROIs assigned P(lateralized) = 0, lateralized ROIs assigned P(lateralized) = 1, and uncertain ROIs assigned P(lateralized) = 0.5. The resulting values were interpolated using the same spatial procedure applied to CLARISA outputs. (**C**) Continuous lateralization probability heatmap produced by CLARISA from ROI-level predicted probabilities. In the heatmaps, warmer colors (red) indicate regions with a higher concentration of lateralized Cx43, whereas cooler colors (blue) indicate regions with a lower concentration of lateralized Cx43. (**D**) MARTA output for the same tissue section. Successfully segmented cardiomyocytes are shown as oriented bounding boxes subdivided into four longitudinal compartments along the principal cell axis, following the original MARTA methodology: the two outer compartments correspond to terminal regions, whereas the two central compartments correspond to lateral regions. CX43 signal is then assigned according to the compartment in which it falls. Tissue regions without a successfully segmented cardiomyocyte remain unshaded, illustrating the difference in spatial coverage between MARTA and the ROI-based approaches. Panels B and C share the same colormap and value range [0, 1].

The continuous lateralization heatmaps generated from CLARISA predictions and from expert annotations showed close agreement in their main spatial structure (see Figure 4). Both approaches identified a prominent warm band running vertically through the central portion of the section, together with colder regions along the upper-left, lower-left, and upper-right borders. Thus, the dominant spatial organization of CX43 lateralization—a predominantly lateralized central axis flanked by more terminal lateral zones—was preserved across the two sources.

Some differences were nevertheless apparent. The expert-derived heatmap reached more extreme local values, most visibly in a saturated warm focus in the lower-central region, where CLARISA produced a warm but less intense response. Conversely, CLARISA produced a broader warm zone along the mid-left margin than the expert annotation. The two maps also differed in texture: the expert-derived heatmap appeared coarser and showed sharper transitions between neighboring regions, whereas CLARISA produced smoother gradients with more diffuse boundaries. This difference is expected from the way the two heatmaps are constructed. The expert heatmap is interpolated from discrete labels, with uncertain regions fixed at P(lateralized) = 0.5, whereas CLARISA uses continuous classifier probabilities. Therefore, differences in smoothness should be interpreted as a consequence of the underlying output format rather than as direct evidence of better or worse biological agreement.

**Figure 5:**
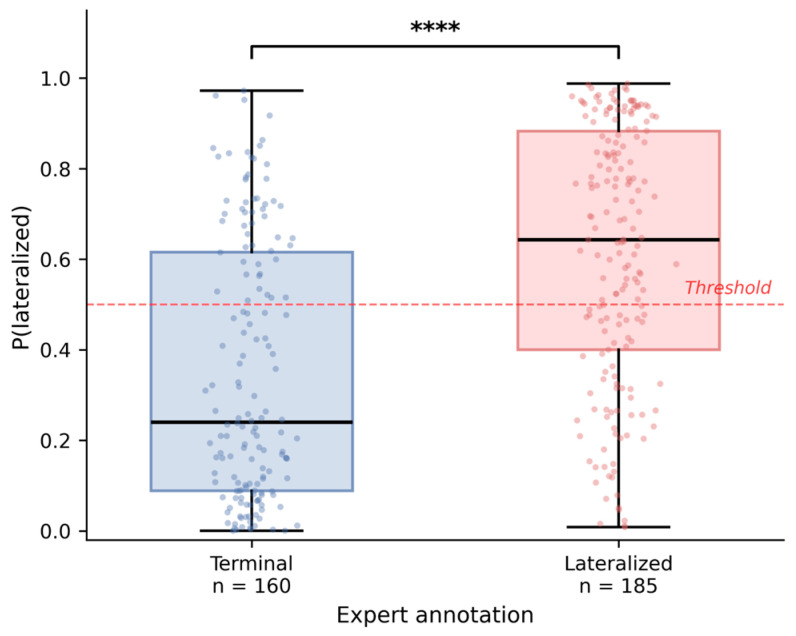
ROI-level distribution of CLARISA-predicted lateralization probabilities grouped by expert annotation. Boxplots show the CLARISA-predicted probability of lateralization, P(lateralized), for the 345 CX43-positive ROIs with a definitive expert label. ROIs are grouped according to whether the expert annotated them as terminal (blue, n = 160) or lateralized (red, n = 185). ROIs labeled as uncertain by the expert were excluded. Boxes indicate the interquartile range and median; whiskers extend to the most extreme non-outlier values. Individual ROIs are overlaid with horizontal jitter. The red dashed line marks the default CLARISA decision threshold, P(lateralized) = 0.5. Statistical significance between expert-terminal and expert-lateralized ROIs was assessed using a two-sided Mann–Whitney U test; **** *p* < 0.0001.

At the ROI level, CLARISA probabilities were broadly aligned with the expert categories, although with substantial overlap between classes (Figure 5). ROIs annotated by the expert as terminal tended to receive lower predicted lateralization probabilities, with a median P(lateralized) of 0.24 and an interquartile range of 0.09–0.62. ROIs annotated as lateralized were shifted toward higher probabilities, with a median of 0.64 and an interquartile range of 0.40–0.88. This distributional shift was statistically significant by a two-sided Mann–Whitney U test (U = 7437.5, *p* = 1.60 × 10^−15^), supporting an association between CLARISA probability scores and expert-defined ROI categories within this section. However, the overlap between distributions indicates that a subset of ROIs received discordant assignments from CLARISA and the expert. The 14 ROIs labeled as uncertain by the expert were excluded from this analysis.

To quantify ROI-level agreement, CLARISA predictions were compared with expert annotations for the 345 ROIs that received a definitive expert label, excluding the 14 ROIs marked as uncertain by the expert. The resulting cross-tabulation is shown in Table 2. Overall percent agreement was 65.5%, with a Cohen’s kappa of 0.31, corresponding to fair-to-moderate agreement. Class-specific agreement was similar for the two categories: 66.9% for terminal ROIs, corresponding to 107 of 160 expert-terminal regions, and 64.3% for lateralized ROIs, corresponding to 119 of 185 expert-lateralized regions.

At the tissue-section level, however, CLARISA and the expert produced highly similar global lateralization estimates. In practical terms, these metrics summarize how much of the detected CX43-positive area was classified as lateralized across the whole section. Two expert-derived values were considered: %LatArea_all_, calculated over all detected CX43-positive ROIs, including those labeled as uncertain, and %LatArea_conf_, calculated only over ROIs for which the expert provided a definitive terminal or lateralized label. CLARISA yielded %LatArea_all_ = 44.26%. The corresponding expert-derived values were %LatArea_all_ = 42.96% and %LatArea_conf_ = 45.20%, with the small difference between the two expert metrics reflecting the 14 ROIs marked as uncertain. The absolute difference between CLARISA and the expert was therefore only 1.30 percentage points when compared with expert %LatArea_all_, and 0.95 percentage points when compared with expert %LatArea_conf_. Because both estimates were computed over the same detected CX43-positive area, this section-level comparison is not confounded by differences in signal coverage. Together, these results indicate that although CLARISA and the expert showed only fair-to-moderate agreement at the level of individual ROIs, their global estimates of CX43 lateralization across the whole section were closely aligned.

#### 2.5.2. Intra-Cellular Comparison with the MARTA Segmentation-Based Method

**Figure 6:**
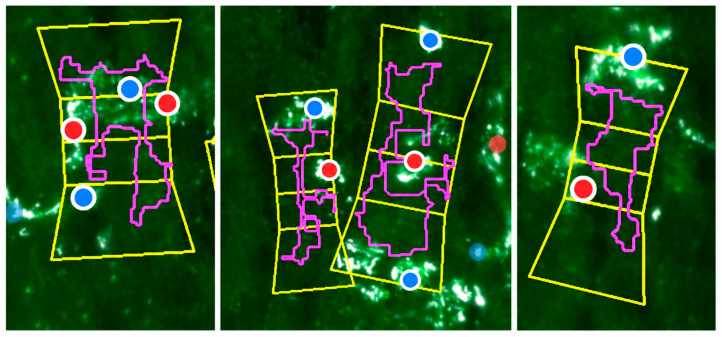
Representative examples of CLARISA-detected ROIs assigned to MARTA compartments. Each panel shows a crop containing a MARTA-segmented cardiomyocyte, represented as an oriented bounding box with internal lateral–terminal partition lines. CLARISA-detected ROI centroids falling within the MARTA bounding box are overlaid as color-coded markers according to the compartment assignment used for the restricted intra-cellular comparison: blue markers indicate ROIs assigned to terminal end compartments, and red markers indicate ROIs assigned to central lateral compartments.

MARTA imposes a different analysis domain from CLARISA because quantification is restricted to the CX43-positive signal located within successfully segmented cardiomyocytes. In IM15, MARTA segmented 82 cardiomyocytes, fewer than the full cardiomyocyte population visible in the tissue section. Consequently, part of the CX43-positive signal detected by CLARISA lay outside any segmented cardiomyocyte and was not included in the MARTA analysis (Figure 4). MARTA also represents CX43 lateralization differently from CLARISA. Rather than assigning a class directly to each CX43-positive ROI, MARTA subdivides each segmented cardiomyocyte into four longitudinal compartments along the principal cellular axis, following the original methodology. The two outer compartments correspond to terminal regions, whereas the two central compartments correspond to lateral regions (Figure 6). CX43 signal was then quantified according to the compartment in which it fell (as described in Section 4.7.2). Within this cell-segmentation-dependent analysis domain, MARTA yielded a mean cell-level percent lateralization of 22.18% ± 26.66% (mean ± SD, as reported by MARTA).

To compare the methods over a shared spatial domain, CLARISA outputs were restricted to the subset of ROIs located within the bounding boxes of the 82 MARTA-segmented cardiomyocytes, according to the inclusion criteria described in Section 4.7.2. A total of 147 of the 359 CLARISA-detected ROIs met this criterion. Among these intra-cellular ROIs, CLARISA classified 55 as lateralized and 92 as terminal, yielding a restricted percent lateralization of 37.4% (Table 3). Among the 139 ROIs in this subset that also had a definitive expert label, the expert classified 68 as lateralized, corresponding to 48.9%. The MARTA estimate remained 22.18% ± 26.66%, since its analysis population was unchanged. Restricting CLARISA to the intra-cellular domain reduced its percent lateralization by 6.9 percentage points relative to the whole-section estimate, from 44.26% to 37.4%. However, an approximately 15 percentage-point difference between CLARISA and MARTA persisted, indicating that the numerical discrepancy between the methods is not explained solely by differences in spatial coverage.

To further characterize this discrepancy at the level of individual ROIs, CLARISA predictions and expert labels were cross-tabulated against the MARTA compartment assignment for each intra-cellular ROI. CLARISA showed 60.5% agreement with MARTA compartment assignments, with a Cohen’s kappa of 0.15 (n = 147; Table 4). The expert showed a similar level of agreement with MARTA, with 58.3% agreement and a Cohen’s kappa of 0.16 among the 139 intra-cellular ROIs with definitive expert labels (Table 5). Eight of the 147 intra-cellular ROIs were labeled as uncertain by the expert; these ROIs were included in the CLARISA–MARTA comparison but excluded from the expert–MARTA comparison.

For reference, CLARISA–expert agreement was also assessed within the same intra-cellular subset. Among the 139 ROIs with a definitive expert label, CLARISA and the expert showed 66.2% agreement, with a Cohen’s kappa of 0.32. This was consistent with the fair-to-moderate agreement observed across the full IM15 section in Section 2.5.1, indicating that restricting the analysis to MARTA-segmented cardiomyocytes did not substantially alter the relationship between CLARISA predictions and expert annotations. Taken together, these results suggest that the lower agreement with MARTA reflects methodological differences in how lateralization is defined and spatially constrained, rather than a CLARISA-specific disagreement with expert interpretation.

## 3. Discussion

### 3.1. Training Data Diversity, Independent Deployment, and Annotation Uncertainty

A key limitation of the present study is the restricted diversity of the development dataset. Although the classifier was trained using transfer learning and a relatively large number of annotated CX43-positive ROIs, these annotations were derived from only two global images and a limited number of tissue sections. These global images corresponded to complete short-axis ventricular sections reconstructed from multiple microscopic fields of view and therefore contained within-section spatial heterogeneity. Nevertheless, the dataset remains limited in terms of the number of independent global images, tissue sections, animals, staining batches, and imaging conditions represented. As a result, the training data may not fully capture the broader variability that can affect CX43 appearance in practice, including differences in section orientation with respect to the myocardial fibers, staining intensity, signal fragmentation, image contrast, blur, or acquisition conditions.

The augmentation strategy used during training partially addressed geometric variability by introducing controlled changes in in-plane orientation, apparent scale, and crop positioning. However, we did not perform a dedicated robustness analysis against realistic imaging distortions such as systematic fluorescence intensity variation, defocus blur, noise, or slice-thickness-related signal degradation. Some of this variability may have been naturally present in the sampled ROIs, but it was not independently controlled or systematically simulated. Therefore, the performance estimates obtained within the training/validation/test framework should be interpreted as preliminary and dataset-dependent, rather than as evidence of broad robustness across heterogeneous imaging conditions.

To provide an additional evaluation beyond the original development images, we analyzed IM15, a section extracted from a global image not used during model development and acquired at a different spatial resolution. This focused whole-section experiment compared CLARISA outputs with expert visual annotation and with the previously published MARTA segmentation-based method. The results support the feasibility of deploying the framework outside the original development images, particularly because CLARISA produced a global percent lateralization estimate closely aligned with the expert annotation. However, this analysis involved a single additional section from a specific experimental context. It should therefore be interpreted as an initial independent deployment evaluation, not as evidence of broad external generalizability.

A further limitation concerns the annotation process used to define the reference labels. The original CX43-positive ROI annotations were generated by a single domain expert, which limits the objective characterization of ground-truth uncertainty. To partially address this issue, we performed a labeling-consistency experiment using the expert annotation workflow described above. As reported in Section 2.3, both repeated annotation by the original expert and independent annotation by a second expert showed substantial agreement with the original labels. However, approximately 15–17% of ROIs changed label across repeated or independent annotation, indicating that a minority of regions remained annotation-sensitive. Visual inspection of these discordant cases suggested that they often corresponded to more challenging regions, including ROIs with weak or poorly defined fiber signal, lower image quality, local blur, fragmented staining, or insufficient contextual information to confidently infer the underlying fiber orientation and, consequently, the distribution of CX43 within it. These observations indicate that annotation uncertainty is not merely a procedural limitation but also reflects the biological and imaging complexity of some regions. Nevertheless, these experiments do not replace a full multi-expert annotation protocol for the complete dataset. Future extensions of the training set should therefore incorporate structured intra- and inter-rater procedures to more rigorously characterize annotation variability.

CLARISA should therefore be understood not only as a fixed classifier trained on the present dataset, but also as a transparent methodological framework that can be inspected, reproduced, and adapted to new experimental settings through additional annotation and retraining. For this purpose, the public repository includes both the full training pipeline and an expert annotation tool, described in Supplementary Section S1.4 and based on the semi-automated ROI detection procedure detailed in Section 4.6.1. This tool is intended to support faster and more standardized expert labeling when expanding the current dataset or generating new datasets under different experimental conditions. By presenting detected ROIs through a standardized interface and exporting structured ROI-level annotations, it may also improve the operational reproducibility of future annotation sessions. In the present work, the tool was used both to annotate IM15 for the focused comparison with expert visual inspection and MARTA, and to support the labeling-consistency experiments described above.

### 3.2. Spatially Mixed Error Patterns and Local Ambiguity

**Figure 7:**
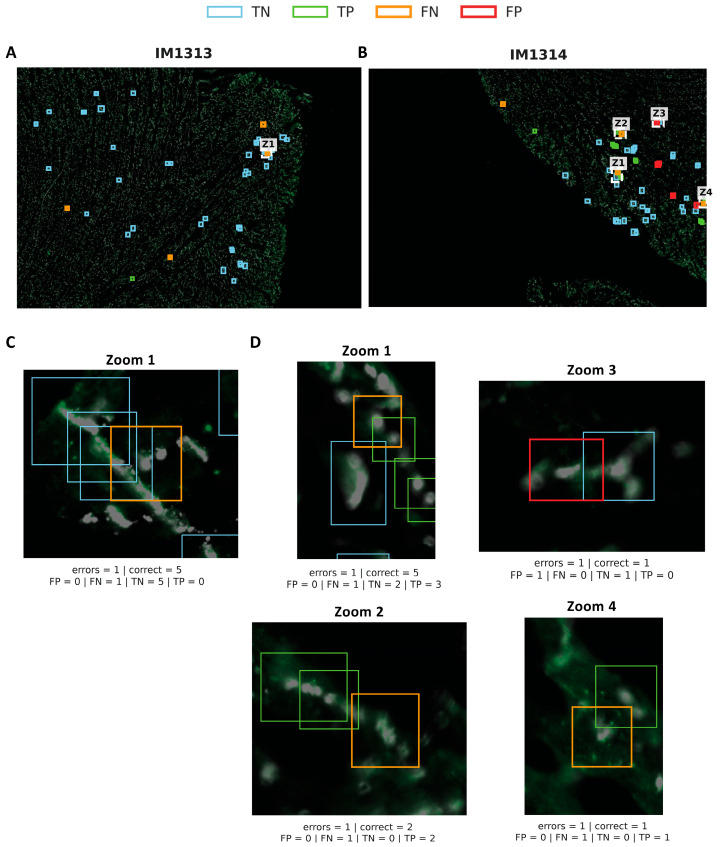
Spatial distribution of ROI-level prediction outcomes in held-out test slides IM1313 and IM1314. ROI-level predictions were mapped back to their original whole-section coordinates and classified relative to the original expert annotation as true negatives (TN, blue), true positives (TP, green), false negatives (FN, orange), and false positives (FP, red). Global views of IM1313 and IM1314 are shown in panels (**A**) and (**B**), respectively. Zoomed regions in panels (**C**,**D**) show locally mixed neighborhoods containing nearby or partially overlapping ROIs with concordant and discordant prediction outcomes. ROI identifiers and bounding-box coordinates for the zoomed examples are listed in Supplementary Table S11.

Spatial inspection of ROI-level predictions on the held-out test tissue slides IM1313 and IM1314 suggested that model errors were not organized into large, homogeneous regions of failure. Instead, both slides showed locally mixed neighborhoods in which correctly and incorrectly classified ROIs coexist (see Figure 7). Thus, the main observation from the spatial analysis was not the presence of fully misclassified image regions, but rather the existence of locally ambiguous microenvironments in which prediction outcomes varied over short spatial distances. Because this analysis was based on two held-out sections, these spatial error patterns should be regarded as descriptive rather than as definitive evidence of general model behavior.

To examine this pattern more closely, we selected regions displaying local coexistence of correct and incorrect predictions, as well as high proximity or partial overlap between ROIs (see Figure 7C,D). The zoomed views show that ROIs located very close to one another, and in some cases partially overlapping, could nevertheless yield different prediction outcomes. This suggests that the classifier may be sensitive to fine local differences in the visual content captured by each ROI. However, the bounding boxes displayed in Figure 7 do not exactly correspond to the full 256 × 256 and 512 × 512 pixel crops used as model input. Therefore, spatial proximity or partial overlap between displayed ROI boxes does not necessarily imply that the model received identical visual information. Conversely, the fact that some partially overlapping ROIs remained correctly classified indicates that shared image content between neighboring crops was not, by itself, sufficient to produce systematic failure. Overall, these observations suggest that the model’s limitations are more closely related to fine-scale local ambiguity than to an inability to recognize larger tissue regions as a whole.

This interpretation is supported by the representative crops shown in Supplementary Section S3. Several discordant or borderline cases occurred in morphologically challenging regions, where the distinction between terminal and lateralized patterns was visually difficult. For example, ROI 3012 in IM1313 and ROIs 3028 and 3034 in IM1314 were better interpreted as borderline examples than as clear-cut classification cases (Supplementary Figures S6 and S7). These ROIs illustrate continuity between well-defined terminal or lateralized patterns and intermediate local morphologies in which the reference label itself may be difficult to assign with full confidence.

Other discordant cases were associated with blur, low contrast, dim signal, or atypical local morphology, as illustrated by ROIs 3011, 3010, 3009, and 3013 in IM1313 and ROIs 3052, 3055, 3063, and 3069 in IM1314 (Supplementary Figures S6 and S7). In selected cases, such as ROI 3069, difficulties in centering the analysis window may also have contributed. This reflects a methodological limitation of the crop-generation procedure: when the bounding box lies close to the image border, or when the relevant signal is not optimally centered within the detected ROI, the corresponding local or contextual crop may not fully capture the surrounding tissue context used by a human observer.

Together with the annotation uncertainty discussed in Section 3.1, these observations suggest that not every model–label disagreement should be interpreted as an unequivocal model failure. Some apparent model errors should therefore be interpreted in light of local ambiguity in the reference label itself, particularly for ROIs located in morphologically complex tissue regions.

### 3.3. Whole-Section Deployment and Methodological Comparison

#### 3.3.1. Deployment on Held-Out Whole Sections

The two held-out sections exhibited distinct whole-section spatial patterns. IM1314 showed more prominent clustered high-probability regions and a higher global lateralization estimate than IM1313, whereas IM1313 displayed a more homogeneous pattern with fewer and more scattered lateralized foci. These observations suggest that CLARISA can generate spatially structured tissue-level outputs, rather than merely producing independent ROI-level classifications.

The ROI-level probability distributions also differed between sections. In IM1313, ROIs assigned to the terminal class were tightly concentrated near zero, whereas ROIs assigned to the lateralized class showed a broader spread of predicted probabilities above the decision threshold. In IM1314, lateralized ROIs were more compactly distributed at high probability values, while terminal ROIs showed broader dispersion. This section-specific confidence structure suggests that CLARISA’s predictions were not equally decisive across both images. In particular, the broader distribution observed in IM1313 is consistent with the local ambiguities discussed in Section 3.2, although this correspondence should be interpreted descriptively rather than as evidence of a general slide-level error pattern.

This analysis should therefore be viewed primarily as a demonstration of whole-section deployment rather than as a formal validation of spatial accuracy. Because exhaustive expert annotation was not available for these held-out sections, its main value lies in showing that CLARISA can be operationally applied to complete tissue sections and can produce interpretable spatial maps and quantitative section-level summaries.

Whole-section deployment should nevertheless be considered semi-automated rather than fully automated. ROI classification is performed automatically by the trained model, but the preceding detection of candidate CX43-positive regions depends on user-defined image-processing parameters, including intensity thresholding and morphological operations. This parameter dependence may influence the number and type of ROIs entering the classifier and, consequently, the estimated global lateralization percentage. However, CLARISA requires substantially fewer user-defined parameters than MARTA, the segmentation-based method used for comparison in this work, which involves more than 25 adjustable parameters. Future work should focus on standardized or automated parameter selection and on systematic robustness analyses across slides with different staining intensity, resolution, contrast, and acquisition conditions.

#### 3.3.2. Exploratory Comparison with Expert Annotation and MARTA in an Independent Section

Whereas Section 2.4 examined whole-section deployment in held-out test sections, Section 2.5 evaluated CLARISA on an additional image not used for model training or hyperparameter tuning. In this independent section, CLARISA outputs were compared with manual expert annotation and with MARTA, a previously published segmentation-based method. At the tissue-pattern level, the expert-derived and CLARISA-derived heatmaps showed similar large-scale spatial organization, including a central warm band and cooler peripheral regions. However, the agreement was not exact, with local differences in the extent and intensity of some regions.

At the ROI level, CLARISA generally assigned lower lateralization probabilities to ROIs annotated by the expert as terminal and higher probabilities to those annotated as lateralized. This supports an association between model output and expert labeling at the level of individual detected regions. However, the overlap around the decision threshold indicates that not all ROIs were cleanly separable into the two categories. Rather than representing only model disagreement, this pattern likely reflects, at least in part, the local ambiguity discussed in Section 3.1 and Section 3.2, where some CX43-positive regions were difficult to assign confidently even by visual inspection.

The comparison with MARTA highlights a methodological distinction that is central to whole-section applicability. MARTA depends on successful cardiomyocyte segmentation and therefore quantifies only the subset of CX43-positive signal located within segmented cells. By contrast, CLARISA operates directly on detected CX43-positive ROIs and is affected by a different set of assumptions, related to ROI detection, crop context, and classification. Accordingly, the comparison should be interpreted as an exploratory methodological benchmark rather than as a definitive test of superiority.

This distinction was reflected in the section-level estimates. In IM15, CLARISA yielded a global lateralization estimate much closer to the expert-derived %LatArea_all_ value than MARTA, with absolute differences of 1.30 and 20.78 percentage points, respectively. However, because CLARISA and MARTA do not quantify identical signal populations, this numerical difference should not be interpreted in isolation. MARTA’s dependence on successful cardiomyocyte segmentation reduced the CX43-positive signal available for quantification in this section, as visible in Figure 4D. Thus, part of the difference between methods reflects their effective analysis domains.

The intra-cellular analysis partially addressed this issue by limiting CLARISA to the 147 ROIs located within MARTA-segmented cardiomyocytes. This reduced CLARISA’s estimate from 44.26% to 37.4%, indicating that spatial coverage contributed to the discrepancy. Nevertheless, an approximately 15 percentage-point difference between CLARISA and MARTA persisted after restriction, while the expert estimate over the same intra-cellular ROIs was 48.9%. This pattern suggests that the numerical gap between methods is not explained solely by spatial coverage, but also by their different operational definitions of lateralization. MARTA computes the fraction of CX43-positive fluorescence area located within geometrically defined lateral compartments of segmented cardiomyocytes, whereas CLARISA and the expert assign labels to individual ROIs based on their visual appearance in context. These definitions can yield different outputs even when applied to the same local CX43 foci.

The ROI-level cross-tabulations support this interpretation. CLARISA and the expert showed similar fair-to-moderate agreement across the full IM15 dataset and within the intra-cellular subset, indicating that restricting the analysis to MARTA-segmented cells did not substantially alter the CLARISA–expert relationship. In contrast, both CLARISA and the expert showed only slight agreement with MARTA’s geometric compartment assignments. This suggests that compartment location captures one aspect of CX43 organization but does not fully reproduce the visual criteria used by the expert or learned by CLARISA from ROI-level annotations.

Overall, this single-section comparison should be regarded as hypothesis-generating. It suggests that CLARISA and segmentation-based approaches provide related but non-equivalent readouts of CX43 lateralization, but systematic evaluation against larger independent datasets and, ideally, biological or functional endpoints will be required to determine their relative validity and use cases.

### 3.4. Biological Relevance and Potential Applications

CX43 is a central mediator of electrical and metabolic coupling in ventricular myocardium, and its redistribution from intercalated discs toward lateral cell membranes has been associated with altered impulse propagation and increased arrhythmogenic vulnerability [20]. However, the quantitative characterization of this topological remodeling and its functional consequences remains a major challenge in the field of cardiac arrhythmias [20].

This spatial component is biologically relevant because changes in CX43 organization may not be captured by bulk expression measurements alone. In several pathological settings, overall CX43 expression assessed by qPCR or Western blot can remain similar despite differences in tissue activation, impulse propagation, or arrhythmia susceptibility [5,21,22]. In such cases, the distribution of CX43 across the myocardium may provide information that complements total expression levels. Methods capable of quantifying CX43 organization over whole tissue sections may therefore help identify structural correlates of conduction disturbances and support comparative studies of connexin remodeling across disease models, treatments, species, or experimental conditions.

A practical motivation for automated analysis is the scale of the annotation task. Manual classification of individual CX43-positive regions as terminal or lateralized is time-intensive; in our workflow, labeling fewer than 250 regions required more than three hours of expert time. By contrast, CLARISA can process thousands of automatically detected regions per image and generate both ROI-level classifications and tissue-scale spatial summaries. This throughput makes it possible to analyze CX43 distribution at a scale that would be impractical through manual inspection alone, particularly in studies involving entire tissue sections or multiple experimental groups.

Within these constraints, CLARISA should be viewed as a proof-of-principle framework rather than a broadly validated or clinically applicable tool. The current evaluation remains limited to one species, a restricted number of tissue sections, and imaging conditions close to those used during model development. Generalization to other species, pathological models, staining protocols, imaging platforms, and acquisition settings will require systematic validation. Nevertheless, by combining segmentation-free ROI detection, multi-scale classification, whole-section probability mapping, and an expert annotation workflow for dataset expansion, CLARISA provides a reproducible and scalable starting point for quantitative studies of CX43 spatial remodeling in experimental cardiac tissue.

## 4. Materials and Methods

### 4.1. Data Acquisition and Histological Imaging

Hearts from 3-month-old male Wistar rats were excised and snap-frozen in liquid nitrogen as previously described [5]. For connexin-43 (CX43) immunodetection, 10-μm cryosections of the left ventricle were washed in phosphate-buffered saline (PBS), fixed in ice-cold methanol, permeabilized with 0.3% Triton X-100 in PBS, and blocked with 1% bovine serum albumin in PBS. Sections were incubated with a primary mouse anti-CX43 antibody (MAB3068, Chemicon International, Temecula, CA, USA) and stained for F-actin (ab112124, Abcam, Cambridge, UK), followed by incubation with a goat anti-mouse FITC-conjugated secondary antibody (111-095-003, Jackson ImmunoResearch, West Grove, PA, USA). Slides were mounted with Vectashield mounting medium (H-1200, Vector Laboratories, Burlingame, CA, USA) and imaged using a Zeiss Axio Imager Z2 microscope equipped with ApoTome.2 (Carl Zeiss, Jena, Germany).

Images used for classifier training, validation, and testing were acquired at a spatial resolution of 0.227 µm/pixel. An additional tissue section, used exclusively for the evaluation described in Section 4.7, was obtained from the same cohort under the same experimental protocol but imaged at a spatial resolution of 0.3 µm/pixel.

### 4.2. Expert Annotation and Dataset Generation of CX43-Positive Regions

**Figure 8:**
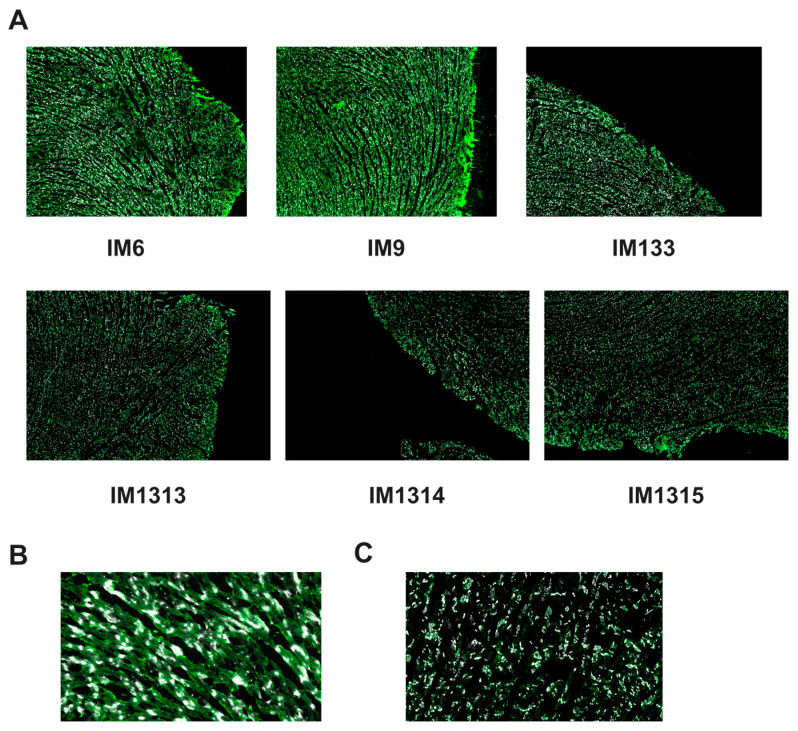
Tissue sections used for expert annotation, with representative close-up views of CX43 staining. (**A**) Overview of the six histological sections from which all annotated CX43-positive regions of interest were drawn. The dataset was partitioned at the section level into training (IM6, IM9, IM133), validation (IM1315), and held-out test (IM1313, IM1314) subsets, as described in Section 4.5.1. All sections were obtained from the left ventricle of 3-month-old male Wistar rats and imaged under the conditions detailed in Section 4.1; green signal corresponds to F-actin and white signal to CX43, and within the myocardium, the black background corresponds to interstitial space. (**B**,**C**) Illustrative magnified views showing the appearance of individual CX43-positive regions at higher zoom, extracted from IM6 (**B**) and IM1313 (**C**). These magnifications are provided for visualization only and were not used as additional ROIs at any stage of model development.

The annotated dataset was constructed from two global fluorescence images, from which six tissue sections were selected for annotation (Figure 8). Each global image corresponded to a complete short-axis ventricular section reconstructed from multiple microscopic fields of view, capturing spatial heterogeneity across the section despite the limited number of global images. Each global image was subdivided into a regular 4 × 3 grid of non-overlapping tissue sections. The six sections selected for annotation occupied different positions within this grid, as indicated in Table 6.

All annotations were performed by a single domain expert according to predefined morphological criteria (Figure 1A). CX43-positive regions were classified as terminal when the signal displayed a morphology and spatial distribution consistent with localization at the longitudinal ends of cardiomyocytes, and as lateralized when the signal exhibited a pattern redistributed along the lateral borders of the cellular profile.

For each annotation, the expert visually identified a CX43-positive region, assigned the corresponding class label, and manually selected a window around it. This window was subsequently processed by a computational pipeline to delineate the CX43-positive area and derive standardized bounding-box coordinates. To this end, the selected window was first converted to grayscale and thresholded at a fixed intensity of 180 on the 8-bit scale to isolate the brightest pixels corresponding to the fluorescent signal. Morphological opening was applied to remove small spurious components, followed by dilation to consolidate the remaining signal area. A bounding rectangle was then fitted around the resulting closed region and expanded by 40 pixels (approximately 9 μm at the imaging resolution reported in Section 4.1) in all directions to reduce the risk of excluding peripheral signal.

In this study, a region of interest (ROI) was defined as a rectangular image patch centered on a CX43-positive region and represented by bounding-box coordinates of the form (x_1_, y_1_, x_2_, y_2_), together with an associated class label. The final dataset comprised 3139 ROIs, of which 1969 were classified as terminal and 1170 as lateralized.

#### Annotation Consistency Assessment

To assess the consistency of the expert annotation used to construct the development dataset, an annotation consistency study was performed on a stratified subset of 180 CX43-positive ROIs sampled from the six annotated tissue sections (30 ROIs per section). Within each section, the subset was built to ensure representation of both classes while maintaining comparability across slides. The final composition of this subset is reported in Supplementary Table S3.

The selected ROIs were then re-evaluated under blinded conditions. First, the same expert who generated the original development dataset performed a repeated annotation session after a substantial time interval had elapsed since the original labeling (12 months), thereby reducing the possibility of recall bias. For each ROI, a 5120 × 5120-pixel context window was extracted from the full-resolution fluorescence image, centered on the ROI bounding-box centroid; the bounding box was indicated by corner markers rendered as a non-destructive canvas overlay. Labels were assigned through a dedicated interactive web interface. This interface was used for the annotation consistency experiment and for the IM15 whole-section comparison described in Section 4.7.1, but not for generation of the original development annotation set. Although the interface retained the same three annotation options available in the original workflow (terminal, lateralized, or uncertain; Supplementary Section S1.4), the expert was explicitly instructed in this consistency experiment to assign each ROI to either the terminal or lateralized class and not to use the uncertain option, so as to preserve direct comparability with the binary development dataset. In addition, a second expert independently annotated the same ROI subset under the same blinded conditions, providing the same inter-observer reference. In both cases, ROIs were presented in randomized order and without disclosure of either the originating tissue section or the original class label.

Agreement between the original and repeated annotation sets was quantified using percent agreement and Cohen’s kappa [23]. In addition, class-specific agreement and the confusion matrix between the original and repeated annotations were computed to characterize the direction of disagreement. Kappa values were interpreted according to standard criteria, with values of 0.41–0.60 considered moderate, 0.61–0.80 substantial, and >0.80 almost perfect agreement [19].

### 4.3. Centered Cropping Extraction and Dual-Scale Input Generation

Each annotated bounding box was transformed into image crops suitable as input to the classifier. The crop center was defined as the integer midpoint of the bounding-box coordinates. Two spatially aligned crops were then extracted around this center: a local crop of 256 × 256 pixels capturing the immediate CX43 neighborhood, and a contextual crop of 512 × 512 pixels providing the surrounding histological context (Figure 1B). At the imaging resolution reported in Section 4.1, these crop sizes enabled simultaneous representation of two complementary scales: the smaller crop preserved sufficient detail to assess the local morphology of the CX43-positive region and its position within the myocardial fiber, whereas the larger crop provided contextual information on CX43 expression in neighboring fibers, following previous multi-scale designs proposed for medical image analysis [24,25].

Together, these two centered views constituted the dual-scale input representation used in the classification framework. Both crops were centered on the same coordinates, ensuring spatial correspondence across scales and consistent positioning of the CX43-positive region within the input, in a manner consistent with the downstream inference pipeline.

### 4.4. Preprocessing and Data Augmentation

After extraction of the local and contextual crops described above, both crop types were resized to 384 × 384 pixels and normalized using the ImageNet channel-wise mean and standard deviation [26]. During training, geometric data augmentation was applied following general recommendations for image augmentation in deep learning pipelines [27]. Specifically, this included horizontal flipping with probability 0.5, vertical flipping with probability 0.2, and a low-amplitude affine transformation with probability 0.3. The affine transformation included translation of up to ±5% in both image axes, isotropic scaling between 0.9 and 1.1, and rotation between −10° and +10°. These transformations were used to introduce controlled variability in in-plane orientation, apparent scale, and minor deviations from the ideal centered configuration, as may occur when three-dimensional myocardial fibers are represented in two-dimensional histological sections, while preserving the centered dual-scale design described above.

For the dual-scale input setting, the same randomly sampled geometric transformation parameters were applied to both synchronized crops to preserve spatial alignment between the local and contextual views. No augmentations were applied during validation or test evaluation, where only resizing and normalization were performed.

No explicit synthetic augmentation of fluorescence intensity, defocus blur, noise, or slice-thickness-related signal degradation was applied.

### 4.5. CX43 Lateralization Classifier

#### 4.5.1. Data Splitting Strategy

The annotated dataset was partitioned at the slide level, such that all ROIs from a given tissue section were assigned to the same subset. This design prevented ROIs from the same section—which share staining conditions, tissue orientation, and background characteristics—from appearing in both training and evaluation subsets, thereby reducing the risk of data leakage. Given the limited number of available sections, a fixed split was adopted: IM6, IM9, and IM133 were assigned to the training set, IM1315 to the validation set; and IM1313 together with IM1314 to the held-out test set. The corresponding ROI counts and class distributions are summarized in Table 7.

Although IM133 originated from the same global image as IM1313, IM1314, and IM1315, it occupied a non-adjacent position within the tissue grid and displayed distinct local tissue characteristics (Table 6). It was therefore considered informative for increasing morphological and staining variability during training, while preserving separation at the section level for validation and testing.

This partitioning strategy resulted in relatively small validation and test sets, and performance estimates should therefore be interpreted with appropriate caution. Nevertheless, slide-level splitting was preferred over ROI-level splitting because it provides a more conservative assessment of generalization. For completeness, ROI-level partitioning was also evaluated, and the corresponding results are reported in Supplementary Section S4.

#### 4.5.2. Model Architecture

The classifier (Figure 1C) operates on the preprocessed dual-scale input described in Section 4.3 and Section 4.4, and outputs a lateralization probability, P(lateralized), through a shared feature extractor followed by a joint classification head.

Feature extraction is performed with an EfficientNetV2-S backbone pretrained on ImageNet [26,28]. The original classification layer is removed, and global average pooling is applied to the final feature map, yielding a 1280-dimensional feature vector for each crop. In the dual-stream configuration, the local and contextual crops are passed sequentially through the same backbone with shared weights, producing two feature vectors f_local_ and f_context_. This shared-weight design ensures that both spatial scales are encoded within a common feature space while reducing the number of trainable parameters relative to architectures with independent backbones.

The two feature vectors are then concatenated to form a fused representation f = [f_local_; f_context_], of dimension 2560, which is fed into the classification head to generate the final prediction (see Figure 1C). Because the prediction is derived from this joint representation, optimization updates the shared backbone using information from both input scales, encouraging the extraction of features that are informative at both the local and contextual levels. Alternative input formulations (local-only and contextual-only) and fusion strategies were also evaluated during architecture optimization (Section 4.5.4).

#### 4.5.3. Training Strategy and Staged Fine-Tuning

Model training was performed using binary cross-entropy with logits as the loss function. To account for class imbalance, the loss was weighted by the inverse class frequency computed on the training set, with the positive-class weight defined as w^+^ = n_0_/n_1_, where n_0_ and n_1_ denote the number of terminal and lateralized samples in the training subset, respectively [29]. This weight was further scaled by a factor of 1.1 to slightly increase the contribution of the minority class during optimization.

Training followed a staged fine-tuning strategy to progressively adapt the pretrained backbone to task-specific morphological features while mitigating catastrophic forgetting [30,31]. In stage 1, the backbone was frozen, and only the classification head was optimized. In stage 2, the last two backbone blocks were unfrozen to allow partial adaptation of high-level features. In stage 3, the entire backbone was unfrozen, and the full network was fine-tuned jointly.

Optimization was performed using AdamW with a weight decay of 1.0 × 10^−4^. A batch size of 16 was used. Training was conducted for 6, 4, and 10 epochs in stages 1, 2, and 3, respectively. Different learning rates were assigned to different parts of the model to allow faster adaptation of the newly initialized classification head while preserving the more general pretrained representations in earlier backbone layers. The classification head was trained with a learning rate of 9.83 × 10^−4^, the last unfrozen backbone block with 3.44 × 10^−4^, and the remaining backbone layers with 1.15 × 10^−4^.

#### 4.5.4. Architecture and Hyperparameter Optimization

Architecture and hyperparameter optimization was performed using Optuna [32] with a Tree-structured Parzen Estimator (TPE) sampler [33]. Optimization proceeded in two sequential stages: an architecture search followed by a fine-tuning search focused on training-related hyperparameters. A detailed description of the optimization framework, including fixed parameters and search spaces, is provided in Supplementary Section S1.1.

In the first stage, alternative model formulations were evaluated while keeping the training configuration fixed. The explored variables included the input representation, the fusion strategy for dual-scale models, the classifier-head type, and the dimensionality and dropout of the classification head. In the second stage, the selected architecture was fixed, and optimization focused on training-related parameters, including dropout, the number of unfrozen backbone blocks during stage 2, the allocation of epochs across the three training stages, the learning-rate hierarchy across model components, and weight decay. The corresponding fixed parameters and search spaces are reported in Supplementary Tables S1 and S2, and the full trial-wise results are provided in Supplementary File S1.

Each trial consisted of training a candidate model using the staged fine-tuning procedure described in Section 4.5.3 while monitoring performance on the validation set. The primary optimization objective was the minimum validation loss achieved during training, and validation AUC was recorded as a complementary metric. Thus, candidate configurations were compared according to their best validation-loss checkpoint rather than according to final-epoch performance.

All trials used the same train–validation split to ensure comparability across configurations. Given the limited dataset size, this procedure should be interpreted as a controlled model selection process rather than an exhaustive exploration of the search space. The best-performing configuration was selected on the basis of minimum validation loss, retrained using the same settings, and the checkpoint with the lowest validation loss in the final run was retained for test evaluation. Further details are provided in Supplementary Section S1.1.3.

#### 4.5.5. Final Evaluation on Held-Out Test Slides

After model selection on the training and validation sets, the final model was evaluated on the held-out test set, which was not used during model development. For each annotated ROI, the model produced a probability of lateralization.

Final classifier performance on the test set was assessed using both threshold-independent and threshold-dependent metrics. Threshold-independent performance was quantified by the area under the receiver operating characteristic curve (ROC-AUC) and the area under the precision-recall curve (PR-AUC, computed as average precision); the latter has been recommended as more informative than ROC-AUC for evaluating binary classifiers on imbalanced datasets [34].

To account for uncertainty in performance estimation on the limited held-out test set, 95% confidence intervals were estimated for ROC-AUC and PR-AUC by stratified bootstrap resampling at the ROI level using 5000 replicates. In each bootstrap replicate, terminal and lateralized ROIs were resampled with replacement within each class and then recombined to generate a bootstrap test set with the same class composition as the original test set. ROC-AUC and PR-AUC were recomputed for each replicate, and 95% confidence intervals were obtained from the 2.5th and 97.5th percentiles of the resulting bootstrap distributions.

Threshold-dependent performance was evaluated at the default decision threshold of 0.5, assigning ROIs to the lateralized class when P(lateralized) ≥ 0.5 and to the terminal class otherwise, and reporting precision and recall for both classes.

### 4.6. Whole-Section Inference Module

#### 4.6.1. Detection of CX43-Positive Regions

The inference pipeline operates on whole tissue sections and therefore begins by identifying candidate CX43-positive regions for subsequent classification (Figure 1D). This detection stage is semi-automated, as it relies on predefined or user-adjusted image-processing parameters. It uses the same morphology-based procedure described for annotation in Section 4.2, but applies it exhaustively to the full image rather than to manually selected windows. Briefly, the image was first converted to grayscale, thresholded at a fixed intensity value, and processed by morphological opening followed by dilation. External contours were then extracted from the resulting binary mask. For each detected contour, a bounding rectangle was computed, isotropically expanded by a fixed margin, and clipped to the image boundaries. The coordinates of all retained bounding boxes were stored for downstream processing.

The detector exposes three adjustable parameters: the intensity threshold used for binarization (*thresh_value*), the size of the structuring element used for morphological opening (*kernel_open*), and the size of the structuring element used for morphological dilation (*kernel_dilate*). Their default values reproduce the configuration used during dataset generation (Section 4.2) and were selected for the staining and imaging conditions described in Section 4.1. Because these operations depend on the visual appearance of the CX43 signal, the detection stage should be considered parameter-dependent; default values may require adjustment when CLARISA is applied to tissue preparations with different staining intensity, background levels, or CX43 expression patterns. Default values and practical guidance for parameter adjustment are provided in Supplementary Section S1.5. A fourth parameter, the bounding-box expansion margin, was kept fixed at 40 pixels throughout this work and typically does not require modification.

#### 4.6.2. ROI Classification and Confidence Thresholding

Each detected ROI was converted into classifier input using the same centered-crop and preprocessing procedure used during training (Section 4.3 and Section 4.4). When CLARISA is applied to images acquired at a spatial resolution different from that of the development set (Section 4.1), the default crop sizes—256 × 256 px for the local crop and 512 × 512 px for the context crop—should be rescaled according to the ratio between the default and target resolutions, in µm/pixel. This preserves the physical field of view represented by each crop.

During inference, the trained classifier produced a lateralization probability, P(lateralized) ∈ [0, 1], for each detected ROI (Figure 1D). The terminal probability was defined as P(terminal) = 1 − P(lateralized). A final class label was then assigned using a confidence threshold τ: ROIs with P(lateralized) ≥ τ were labeled as lateralized, ROIs with P(lateralized) ≤ 1 − τ were labeled as terminal, and the remaining ROIs were labeled as indeterminate. With the default value τ = 0.5, every ROI receives a definitive label because the two decision regions cover the full probability interval. Values of τ > 0.5 introduce an indeterminate band around 0.5, reducing coverage but increasing confidence in the assigned labels. For each ROI, the classifier output included the bounding-box coordinates, predicted class probabilities, and final assigned label.

#### 4.6.3. Spatial Probability Maps and Classification Overlays

For each tissue section, the inference module generated two complementary spatial representations of the predicted CX43 lateralization pattern (Figure 1E): a continuous probability map and a discrete classification overlay.

The continuous probability map was generated by spatially smoothing the per-ROI lateralization probabilities across the tissue section. Briefly, ROI-level probabilities were interpolated using a Gaussian-weighted Nadaraya-Watson kernel estimator [35,36], producing a field, H(x,y), that represents the local predicted probability of lateralization at each tissue location. Values close to 1 indicate regions dominated by nearby ROIs predicted as lateralized with high confidence, whereas values close to 0 indicate regions dominated by terminal predictions. The Gaussian bandwidth, σ, determines the spatial scale of the heatmap and was set adaptively from the observed ROI density of each image using a neighborhood parameter, k. This parameter controls the approximate number of nearby ROIs contributing to the local estimate; the full mathematical derivation and parameter guidance are provided in Supplementary Section S1.6. For all images analyzed in this study, k = 12 was used, and the corresponding σ values are reported in Table 8. Heatmap-based metrics defined in Supplementary Section S1.3 therefore depend on σ and k, whereas the primary area-based metrics defined in Section 4.6.4 are computed directly from the discrete per-ROI classifications and are independent of k.

The second output was a discrete classification overlay, generated by filling each classified ROI contour with a color corresponding to its assigned label: terminal, lateralized, or indeterminate. Indeterminate ROIs, defined by the confidence rule described in Section 4.6.2, were shown with a distinct color to facilitate visual inspection. This overlay provides a direct visualization of the per-ROI decisions and complements the continuous probability map. Both outputs were exported as image files, and the continuous probability map was also saved as a color overlay blended with the original histological image.

#### 4.6.4. Whole-Section Percent Lateralization Metrics

For each processed tissue section, the inference module computed global metrics summarizing the fraction of detected CX43-positive area classified as lateralized. These metrics were designed to provide section-level readouts of the ROI classifications, rather than absolute physiological measurements independent of the detection settings.

Three masks were defined from the detected ROI contours. Ω_all_ represented the union of all detected CX43-positive ROI contours. Ω_conf_ represented the subset of Ω_all_ corresponding to ROIs that received a confident label, excluding indeterminate ROIs. Ω_lat_ represented the subset of Ω_conf_ corresponding to ROIs assigned to the lateralized class. The primary area-based metrics were then defined as:

$${\%LatArea}_{all}=100\times \frac{\left|\Omega_{lat}\right|}{\left|\Omega_{all}\right|}$$

$${\%LatArea}_{conf}=100\times \frac{\left|\Omega_{lat}\right|}{\left|\Omega_{conf}\right|}$$

where |·| denotes pixel area. %LatArea_all_ represents the lateralized fraction over the entire detected CX43-positive area and therefore includes indeterminate ROIs in the denominator. By contrast, %LatArea_conf_ restricts the denominator to ROIs with a confident terminal or lateralized label, providing an estimate based only on regions for which the classifier committed to a decision. Both metrics were computed directly from the classification overlay described in Section 4.6.3 and therefore depended only on the discrete per-ROI labels and the geometry of the detected contours. They are independent of the heatmap smoothing parameter *k*.

As complementary outputs, two heatmap-based metrics, %LatHeat_all_ and %LatHeat_conf_, were computed as the pixel-wise mean of H(x,y) over the same masks. These metrics summarize lateralization in a confidence-weighted manner, allowing each pixel to contribute according to the propagated probability from the continuous heatmap rather than a hard class label. Across all analyzed sections, the heatmap-based values remained within 5–6 percentage points of the corresponding area-based metrics. Full definitions and per-section values for these complementary metrics are reported in Supplementary Section S2.2.

### 4.7. Whole-Section Deployment: Comparison with Expert Annotation and MARTA

To assess the behavior of the complete inference pipeline in a representative deployment scenario, image IM15 was analyzed using three independent approaches: CLARISA, manual annotation by a domain expert, and the previously published segmentation-based methodology MARTA. IM15 was extracted from a global image not used during model development and was acquired at a slightly different spatial resolution from the development set (0.3 µm/pixel vs. 0.227 µm/pixel; Figure 4A). This analysis therefore provided an initial assessment of whole-section inference under acquisition conditions not encountered during training.

Because IM15 differed from the development images in both spatial resolution and CX43 signal appearance, the inference parameters were adjusted before deployment. Resolution-dependent crop sizes were rescaled to preserve the physical field of view represented by each crop, and the three detection parameters were jointly adjusted to account for the weaker and more spatially fragmented CX43 signal observed in this section. The adjusted values are summarized in Table 8, and the rationale for each modification is provided in Supplementary Section S1.7. These adjustments reflect the semi-automated nature of the current whole-section deployment workflow, which requires user supervision when imaging characteristics differ from those of the development set.

#### 4.7.1. Expert Annotation and CLARISA–Expert Agreement

For the whole-section comparison study, IM15 was independently analyzed by a domain expert using a custom in-house interactive annotation tool. The expert was the same individual who generated the training dataset described in Section 4.2. The tool was designed to facilitate ROI-level labeling of regions detected by the ROI detection pipeline described in Section 4.6.1. Additional details on the interface are provided in Supplementary Section S1.4.

For each detected region, the expert assigned one of three labels: terminal, lateralized, or uncertain. The uncertain label was used when the region could not be confidently assigned to either terminal or lateralized. To generate expert-derived spatial outputs and global metrics, these manual labels were passed through the same downstream functions used by CLARISA for probability map construction and global quantification (Section 4.6.3 and Section 4.6.4). Terminal regions were assigned P(lateralized) = 0, lateralized regions were assigned P(lateralized) = 1, and uncertain regions were assigned P(lateralized) = 0.5. At the default threshold τ = 0.5, uncertain regions were treated as indeterminate: they contributed to the total detected CX43-positive area but were excluded from the confident terminal and lateralized subsets. This procedure represents the manual counterpart of the CLARISA workflow for researchers wishing to quantify CX43 lateralization without automated classification.

ROI-level agreement between CLARISA and the expert was assessed using only ROIs with a definitive expert label; ROIs labeled as uncertain were excluded. In addition to binary agreement analysis, the distributions of CLARISA-predicted P(lateralized) were compared between ROIs labeled by the expert as terminal and lateralized using a two-sided Mann–Whitney U test. Agreement was summarized by percent agreement and Cohen’s kappa [23], with class-specific agreement computed separately for terminal and lateralized ROIs. Kappa values were interpreted according to the criteria described in Section Annotation Consistency Assessment [19]. Section-level comparison was based on the area-based metrics defined in Section 4.6.4, computed for both CLARISA and the expert annotation. Because CLARISA was applied using the default threshold τ = 0.5, no ROIs were assigned to the indeterminate category; therefore, %LatArea_conf_ coincided with %LatArea_all_ for CLARISA and was not reported separately.

#### 4.7.2. Intra-Cellular Comparison with the MARTA Segmentation-Based Method

IM15 was additionally processed with MARTA, a previously published segmentation-based methodology, using the parameterization selected for the imaging conditions of this study. MARTA input parameters were selected by the same domain expert who generated the IM15 annotations, after iterative review of intermediate segmentation outputs; the final parameter values are reported in Supplementary Section S1.8. Because CLARISA, the expert annotation workflow, and MARTA quantify partially different populations of CX43 signal, section-level numerical comparison was restricted to the area-based metrics defined in Section 4.6.4. Heatmap-based metrics were not considered for this comparison because they are not meaningful when derived from binary expert labels. CLARISA was applied using the default decision threshold τ = 0.5; under this setting, no ROIs were assigned to the indeterminate category, and %LatArea_conf_ therefore coincided with %LatArea_all_ for CLARISA.

To compare the methods over a shared spatial domain, CLARISA-detected ROIs were matched to MARTA-segmented cardiomyocytes. An interactive browser-based tool was developed for this purpose. The MARTA output image, including cardiomyocyte bounding boxes and lateral–terminal partition lines, was used as the background, and CLARISA ROI centroids were overlaid as color-coded markers. The observer reviewed each ROI and assigned a MARTA compartment label when the ROI could be unambiguously located within a segmented cardiomyocyte, as can be seen in Figure 6.

An ROI was included in the intra-cellular subset if more than 50% of its CX43-positive area fell within a single MARTA cardiomyocyte bounding box and its position was clearly within either the lateral or terminal compartment of that cell. ROIs were excluded if they fell outside all MARTA bounding boxes or if their CX43-positive area straddled a lateral–terminal boundary without a clear compartment assignment. This annotation was performed in a single session by the same observer who generated the expert annotation of IM15.

For each included ROI, the MARTA compartment label was assigned according to its location within the segmented cardiomyocyte. ROIs in the terminal, intercalated-disc compartments at the longitudinal ends of the bounding box were assigned the terminal class, whereas ROIs in the central lateral compartments were assigned the lateralized class. CLARISA’s binary prediction for the same ROI was taken directly from the inference output at τ = 0.5, and expert labels were taken from the annotation session described in Section 4.7.1. Pairwise agreement between CLARISA predictions, expert labels, and MARTA compartment assignments was quantified by cross-tabulation, percent agreement, and Cohen’s kappa. ROIs labeled as uncertain by the expert were excluded from comparisons involving the expert. Finally, the MARTA cell-level percent lateralization metric, %CxLat2, is an area-weighted metric computed over segmented cellular compartments and is therefore not directly comparable to the ROI-based estimates produced by CLARISA and the expert annotation workflow.

## 5. Conclusions

In this study, we developed CLARISA, a segmentation-free, ROI-based framework for assessing CX43 lateralization in fluorescence images of ventricular myocardium without requiring explicit cardiomyocyte segmentation. An expert-annotated dataset of CX43-positive ROIs classified as terminal or lateralized was generated to support model training, and a dual-scale transfer-learning classifier was developed to predict the lateralization status of individual regions from their local morphology and surrounding tissue context. The classifier was integrated into a semi-automated whole-section inference workflow that generates spatial probability maps and quantifies the proportion of lateralized CX43 signal across complete tissue images.

The classifier was integrated into a semi-automated whole-section inference workflow that generates spatial probability maps and estimates the proportion of lateralized CX43 signal across complete tissue images. The present results support the feasibility of automated ROI classification and whole-section spatial mapping as a proof-of-principle methodological framework. However, CLARISA should not yet be interpreted as a fully automated, broadly generalizable, or clinically validated tool. The current evidence remains limited by the size and diversity of the dataset, the reliance on annotations initially generated by a single expert, the need for manual adjustment of some inference parameters, and the limited validation across heterogeneous imaging conditions.

Beyond the trained classifier, CLARISA is provided as a transparent and extensible framework. The pretrained model, annotation resources, expert annotation tool, and training scripts are publicly available, allowing users to inspect and reproduce the workflow, evaluate the model on comparable data, and generate new ROI-level datasets for dataset-specific adaptation.

Future work should include larger and more diverse datasets, structured multi-expert annotation protocols, automated or standardized parameter selection, and broader validation across independent tissue preparations, species, staining protocols, pathological models, and imaging pipelines. Overall, CLARISA provides a scalable starting point for segmentation-free CX43 lateralization assessment in experimental cardiac tissue, particularly in studies where manual ROI-level quantification would be labor-intensive and difficult to scale.

## Figures and Tables

**Table 1:** Whole-section inference results on held-out test sections.

Section	N Detected ROIs	Terminal ROIs	Lateralized ROIs	% LatArea_all_
IM1313	3715	3471	244	6.63%
IM1314	1233	948	285	19.06%

**Table 2:** Cross-tabulation of CLARISA predictions and expert annotations in image IM15. Only ROIs with a definitive expert label were included. Fourteen ROIs labeled as uncertain by the expert were excluded. Overall percent agreement = 65.5%; Cohen’s kappa = 0.31.

	Expert: Terminal	Expert: Lateralized	Total
CLARISA: Terminal	107	66	173
CLARISA: Lateralized	53	119	172
Total	160	185	345

**Table 3:** Restricted percent lateralization estimates within the MARTA-segmented intra-cellular domain. For CLARISA and the expert, estimates were computed using only CLARISA-detected ROIs located within MARTA-segmented cardiomyocyte bounding boxes. For the expert, 8 of the 147 intra-cellular ROIs were labeled as uncertain and were excluded from the definitive-label estimate. MARTA reports a cell-level area-weighted percent lateralization metric, whereas CLARISA and expert estimates are ROI-based; therefore, the values are compared as complementary summaries rather than identical measurement endpoints.

Method	Percent Lateralization	n (Analysis Unit)
MARTA (cell-level)	22.18% ± 26.66% (mean ± SD)	82 cardiomyocytes
CLARISA (restricted)	37.4%	147 intra-cellular ROIs
Expert (restricted)	48.9%	139 intra-cellular ROIs

**Table 4:** Cross-tabulation of CLARISA predictions and MARTA compartment assignments for the 147 intra-cellular ROIs. Percent agreement = 60.5%; Cohen’s kappa = 0.15.

	MARTA: Terminal	MARTA: Lateralized	Total
CLARISA: Terminal	64	28	92
CLARISA: Lateralized	30	25	55
Total	94	53	147

**Table 5:** Cross-tabulation of expert labels and MARTA compartment assignments for the 139 intra-cellular ROIs with a definitive expert label. Eight ROIs labeled as uncertain by the expert were excluded. Percent agreement = 58.3%; Cohen’s kappa = 0.16.

	MARTA: Terminal	MARTA: Lateralized	Total
Expert: Terminal	51	38	89
Expert: Lateralized	20	30	50
Total	71	68	139

**Table 6:** Distribution of annotated CX43-positive regions across global images and tissue sections.

Global Image	Tissue Section	Grid Position	Terminal CX43-Positive Regions	Lateralized CX43-Positive Regions
Global 1	IM9	row 2, col 3	1161 (77.19%)	343 (22.81%)
Global 1	IM6	row 3, col 3	567 (42.06%)	781 (57.94%)
Global 2	IM133	row 1, col 3	110 (96.49%)	4 (3.51%)
Global 2	IM1313	row 3, col 3	43 (89.58%)	5 (10.42%)
Global 2	IM1314	row 4, col 1	38 (66.67%)	19 (33.33%)
Global 2	IM1315	row 4, col 2	50 (73.53%)	18 (26.47%)

**Table 7:** Dataset partitioning at the slide level.

Subset	Sections	n (ROIs)	Class Terminal (%)	Class Lateralized (%)
Training	IM6, IM9, IM133	2966	61.97	38.03
Validation	IM1315	68	73.53	26.47
Test	IM1313, IM1314	105	77.14	22.86

**Table 8:** Inference parameters used for the IM15 deployment evaluation. Resolution-dependent crop sizes were rescaled to preserve the physical field of view described in Section 4.6.2. The neighborhood parameter k was kept at its default value; the reported Gaussian kernel standard deviation σ, is a derived quantity recomputed from the observed ROI density of IM15, as described in Section 4.6.3. Detection parameters were adjusted jointly to account for the weaker and more spatially fragmented CX43 signal observed in the evaluation section. The detailed rationale for these adjustments is provided in Supplementary Section S1.7.

Parameter	Development Set	IM15	Reason for Adjustment
Spatial resolution	0.227 µm/pixel	0.3 µm/pixel	Acquisition condition
crop_local	256 px	192 px	Rescaled to preserve physical FOV (Section 4.6.2)
crop_context	512 px	384 px	Rescaled to preserve physical FOV (Section 4.6.2)
σ (derived, in px)	126 px	94 px	Recomputed from observed ROI density (Section 4.6.3)
thresh_value	180	150	Weaker CX43 staining on the evaluation section
kernel_open	9	5	Reduced to avoid eliminating genuine low-intensity signal
kernel_dilate	5	21	Increased to reconnect spatially fragmented CX43-positive regions
τ (decision threshold)	0.5	0.5	Default retained

## Data Availability

The source code used in this study is publicly available in the project GitHub repository: https://github.com/dgattari/CLARISA.git v1.0.0 (accessed on 25 May 2026). The pretrained model checkpoint is publicly available on Hugging Face: https://huggingface.co/jsanchoz/clarisa-cx43-classifier (accessed on 25 May 2026). The fluorescence images and annotated ROIs used in this study are publicly available through Zenodo: https://doi.org/10.5281/zenodo.19664101 (accessed on 25 May 2026). Additional implementation details, including instructions for reproducing the pipeline, applying the pretrained model, training from scratch on new data, and using the expert annotation tool, are provided in the public code repository.

## Supplementary Materials

The following supporting information can be downloaded at: https://www.mdpi.com/article/10.3390/ijms27115033/s1.
