# Supplementary material for: CLARISA: Connexin-43 Lateralization Automated ROI-Based Image Signal Analyzer
Source: Int J Mol Sci. 2026 Jun 2;27(11):5033. doi: 10.3390/ijms27115033 (PMC13256817; doi:10.3390/ijms27115033)
Supplement: Supplementary file 1 [file ijms-27-05033-s001.zip › ijms-4301630-supplementary.pdf]

Supplementary Data for "CLARISA: Connexin-43 Lateralization Automated ROI-based Image Signal Analyzer"

Daniel Gattari[1], Joseba Sancho-Zamora[2], Debora Chan[1], Emiliano Diez[3], Mariano Llamedo Soria[4], Mario Rossi[1y5]

[1] Faculty of Engineering, Austral University, Pilar, Buenos Aires, Argentina
[2] Tecnun School of Engineering, Universidad de Navarra, Donostia, Spain
[3] Institute of Experimental Medicine and Biology of Cuyo (IMBECU), CONICET, Mendoza, Argentina
[4] Electronics Department, National Technological University, Buenos Aires, Argentina
[5] Functional Genomics and Data Science, Institute for Translational Medicine Research (IIMT), CONICET-Universidad Austral, Pilar, Buenos Aires, Argentina

S1. Supplementary Methods

S1.1. Hyperparameter and Architecture Optimization

This section details the search spaces, fixed parameters, and model-selection criteria used in the two-stage Optuna optimization procedure summarized in Section 4.5.4 of the main manuscript.

S1.1.1. Architecture Search

In the first optimization stage, alternative model formulations were compared while keeping the training configuration fixed. The explored variables included the input representation, the fusion strategy used when both spatial scales were combined, the classifier-head type, and the dimensionality and regularization of the classification head. Three input representations were evaluated: local-only (256 × 256 crop); contextual-only (512 × 512 crop); and dual-scale, combining both views. For dual-scale configurations, two fusion strategies were considered: dual-stream (shared weights), in which local and contextual crops were processed by parallel branches sharing the same backbone parameters and fused at the feature level; and channel-wise concatenation. Classifier-head variants included an MLP head and a logistic head. All training-related parameters not explicitly optimized in this stage were kept fixed (Supplementary Table S1). The architecture search comprised 14 trials; full trial-wise results are provided in Supplementary File 1 (architecture).

Supplementary Table S1. Architecture search: fixed parameters and search space.

Category	Parameter	Value / Range
Fixed	Stage 1 epochs	5
Fixed	Stage 2 epochs	5
Fixed	Stage 3 epochs	8
Fixed	Unfrozen backbone blocks in Stage 2	1
Fixed	Head learning rate	1.0×10^{-3}
Fixed	Last-block learning rate	3.0×10^{-4}
Fixed	Remaining-backbone learning rate	1.0×10^{-4}
Fixed	Weight decay	1.0×10^{-4}
Fixed	Positive-class scaling factor	1.1
Fixed	Decision threshold	0.5
Search space	Input representation	Local-only, Contextual-only, Dual-scale
Search space	Fusion strategy (dual-scale only)	Dual-stream (shared weights), Channel-wise concatenation
Search space	Classifier-head type	MLP head, Logistic head
Search space	Hidden dimension	64, 128, 256
Search space	Dropout	0.3, 0.5

S1.1.2. Fine-Tuning Search

In the second optimization stage, the selected architecture was fixed and optimization focused on training-related hyperparameters. The fixed architecture corresponded to the dual-scale input formulation combined with dual-stream (shared weights) fusion and an MLP head with 256 hidden units. The head learning rate was optimized directly; the effective learning rates for the last unfrozen backbone block and the remaining backbone layers were obtained by

multiplying the head learning rate by the corresponding last-block LR ratio and remaining-backbone LR ratio. All fixed and variable parameters are reported in Supplementary Table S2. The fine-tuning search comprised 100 trials; full trial-wise results are provided in Supplementary File 1 (fine-tuning).

Supplementary Table S2. Fine-tuning search: fixed parameters and search space.

Category	Parameter	Value / Range
Fixed	Input representation	Dual-scale
Fixed	Fusion strategy	Dual-stream (shared weights)
Fixed	Classifier-head type	MLP head
Fixed	Hidden dimension	256
Fixed	Input resize	384 × 384
Fixed	Positive-class scaling factor	1.1
Fixed	Decision threshold	0.5
Search space	Dropout	0.3, 0.4, 0.5
Search space	Head learning rate	1.0×10^{-4} to 1.0×10^{-3} (log scale)
Search space	Last-block LR ratio	0.2, 0.3, 0.35, 0.5
Search space	Remaining-backbone LR ratio	0.1, 0.2, 0.333, 0.35
Search space	Stage 1 epochs	4, 6, 8
Search space	Stage 2 epochs	4, 6, 8
Search space	Stage 3 epochs	8, 10, 12
Search space	Unfrozen backbone blocks in Stage 2	1, 2, 3
Search space	Weight decay	1.0×10^{-4}, 3.0×10^{-4}, 1.0×10^{-3}

S1.1.3. Optimization Objective and Model Selection

Each trial consisted of training a candidate model on the training set using the staged fine-tuning procedure described in Section 4.5.3 of the main manuscript, while monitoring performance on the validation set. The primary optimization objective was the minimum validation loss achieved during training; validation AUC was recorded as a complementary metric. All trials within a given optimization stage used the same train–validation split, ensuring direct comparability across configurations. The best-performing configuration was selected according to the minimum validation loss. The final model was then retrained using this configuration, and the checkpoint with the lowest validation loss was retained for test evaluation.

S1.2. ROI Subset Used for the Annotation Consistency Study

The detailed composition of the 180-ROI subset used for the annotation consistency study is summarized in Supplementary Table S3. For tissue sections containing fewer than 15 lateralized ROIs, all available lateralized ROIs were included, and the remaining quota was completed with terminal ROIs.

Supplementary Table S3. Composition of the ROI subset used for the annotation consistency study, based on the original annotation labels.

Tissue section	Terminal ROIs	Lateralized ROIs	Total ROIs
IM1313	25	5	30
IM1314	15	15	30
IM1315	15	15	30
IM133	26	4	30

IM6	15	15	30
IM9	15	15	30
Total	111	69	180

S1.3. Definition of Heatmap-Based Metrics

In addition to the area-based metrics defined in Section 4.6.4 of the main text, two complementary heatmap-based metrics were computed from the continuous lateralization probability field H(x, y) described in Section 4.6.3 and Supplementary Section S1.6. Unlike the area-based metrics, which are derived from the discrete per-ROI labels, these metrics summarize the continuous probability map by weighting each pixel according to the prediction confidence propagated from nearby classified regions:

$$\%LatHeat_{all} = \frac{100}{\Omega_{all}} \sum_{(x,y)\in\Omega_{all}} H(x,y)$$

$$\%LatHeat_{conf} = \frac{100}{\Omega_{conf}} \sum_{(x,y)\in\Omega_{conf}} H(x,y)$$

where $\Omega_{a}\ell\ell$ and Ω^{conf} are defined as in Section 4.6.4, and the summation runs over all image pixels within each mask. Unlike their area-based counterparts, these heatmap-based metrics depend on the smoothing parameter σ of the Gaussian kernel (equivalently, on the parameter k from which σ is derived; see Supplementary Section S1.6). They provide a complementary view in which ROIs with intermediate predicted probabilities contribute partially rather than being forced into a binary assignment.

S1.4. Expert Annotation Tool

The expert annotation tool used to generate reference annotations for the whole-section comparison (Section 4.7 of the main manuscript) was implemented as a lightweight local web application. At launch, the tool loads the target tissue section, runs the ROI detection pipeline described in Section 4.6.1 with the same configuration later used at inference time, and displays the image in a navigable canvas with each detected CX43-positive region marked by a dot at its centroid. Clicking on a dot opens a dialog that shows two co-centered crops of the selected region — a local view and a wider contextual view — and exposes three labelling options: terminal, lateralized, and uncertain. Keyboard shortcuts are available for each option, as well as for undoing the last annotation and for dismissing the dialog without labelling. Annotations are saved progressively to a CSV file so that a session can be interrupted and resumed without loss of state.

Supplementary Figures S1 and S2 show, respectively, the main annotation canvas and the per-ROI annotation dialog.

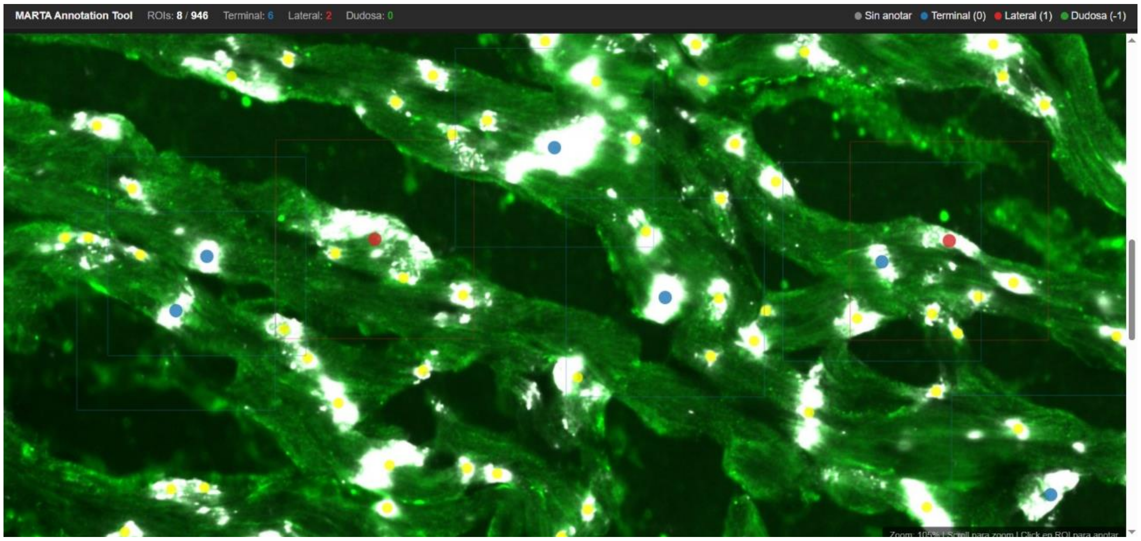

Supplementary Figure S1. Main annotation canvas of the interactive expert annotation tool. Screenshot of the navigable canvas on which the expert performs annotation. The tool has automatically detected all candidate CX43-positive regions using the ROI detection pipeline described in Section 4.6.1 of the main text, and each detected region is marked by a dot at its centroid. Dots are color-coded by assigned class (terminal, lateralized, or uncertain); unannotated regions are shown in a neutral color. The toolbar reports running counts of total detected regions, annotated regions, and annotations per class.

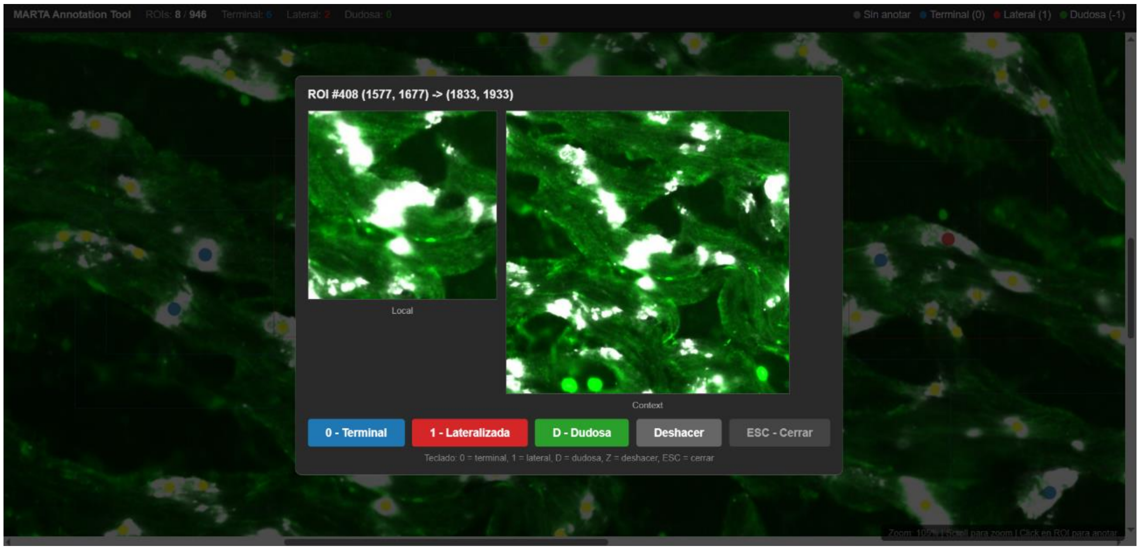

Supplementary Figure S2. Per-ROI annotation dialog of the interactive expert annotation tool. Screenshot of the dialog opened when the expert clicks on a detected region. Two co-centered crops are displayed side by side: a local view (256 × 256 px) and a wider contextual view (512 × 512 px). Three labelling options are exposed — terminal, lateralized, and uncertain — together with controls for undoing the last annotation and dismissing the dialog. Keyboard shortcuts are indicated at the bottom of the dialog.

S1.5. Detection Parameter Defaults and Practical Adjustment Guidance

This section documents the default values and practical guidance for the three adjustable parameters of the CX43-positive region detector described in Section 4.6.1 of the main text. The defaults were selected for the staining and imaging conditions described in Section 4.1; they may require adjustment when CLARISA is applied to images with different staining intensity, background levels, or CX43 expression patterns. A fourth parameter, the bounding-box expansion margin, was kept fixed at 40 pixels throughout this work and typically does not require modification.

Supplementary Table S4. Adjustable parameters of the CX43-positive region detector and practical guidance for tuning.

Parameter	Role	Default	Practical guidance for adjustment
**thresh_value**	Intensity threshold (0–255) used to binarize the grayscale image. Pixels above this value are considered CX43-positive.	180	Decrease (e.g., 100–150) if staining is weak or the image is dark; increase (e.g., ≥200) if staining is strong or background noise is high.
**kernel_open**	Size (px) of the square structuring element used for morphological opening. Removes isolated bright regions smaller than the kernel.	9	Decrease when CX43 expression is low or fragmented to preserve genuine low-intensity signal; increase when staining is strong and small detections are expected to be noise. Set to 1 to disable. Note: this operation is irreversible — signal removed at this step cannot be recovered by subsequent operations.
**kernel_dilate**	Size (px) of the square structuring element used for morphological dilation. Expands detected regions and can merge nearby detections into a single ROI.	5	Increase when CX43 signal is fragmented or sparse so that biologically coherent regions reconnect into single ROIs; decrease if distinct ROIs are being merged incorrectly. Set to 1 to disable.

S1.6. Spatial Probability Map: Mathematical Derivation and Bandwidth Parameter

The continuous probability map described in Section 4.6.3 of the main text was computed as follows. For each classified ROI, the predicted probability P(lateralized) was added to an accumulator map P at the position of the ROI centroid; both P and a parallel weight map W, of the same spatial dimensions as the input image and initialized to zero, were used to track the accumulated probability mass and the number of contributions received at each location. Both maps were then convolved with a two-dimensional Gaussian kernel $G\sigma$ of standard deviation σ, and the interpolated probability field H(x,y) was computed as:

$$H(x,y) = \frac{(P * G_\sigma)(x,y)}{\max\{(W * G_\sigma)(x,y),\ \varepsilon\}}$$

where $*$ denotes two-dimensional convolution and $\varepsilon = 10^{-8}$ prevents division by zero in regions without contributing ROIs. This formulation corresponds to a Gaussian-weighted local average of the ROI-level probabilities and can be interpreted as a Nadaraya–Watson kernel estimator [30,31] applied to the sparse set of classified regions. The resulting field H(x,y) represents, at each tissue location, a spatially smoothed estimate of the local lateralization probability: values near 1 indicate areas surrounded by ROIs predicted as lateralized with high confidence; values near 0 indicate areas dominated by terminal signal.

The standard deviation σ was set based on the local density of detected ROIs so that the effective integration window of the kernel contained approximately k neighbouring ROIs:

$$\sigma = \sqrt{\frac{k}{4\pi\rho}}$$

where ρ is the ROI density (number of detected regions divided by the tissue area) and k is the expected number of ROIs within a disk of radius 2σ. For the development-set images (0.227 µm/pixel), $\rho \approx 1.2 \times 10^{-3}$ ROIs/µm^2; setting k = 12 yields σ ≈ 29 µm (126 px). For IM15 (0.3 µm/pixel), the same formula yields σ = 94 px. With σ ≈ 29 µm, the effective kernel area $4\pi\sigma^2 \approx$ 10,500 µm^2 encompasses several adjacent cardiomyocytes, a scale consistent with detecting tissue zones in which lateralization is shared among neighbouring myocytes.

In this scheme, k is the primary hyperparameter controlling the spatial scale of the heatmap; σ is a deterministic function of k and the observed ROI density. Values of k in the range 10–15 are recommended: larger values produce smoother maps; smaller values produce more localized maps. Note that k affects only the continuous probability map and the complementary heatmap-based metrics defined in Supplementary Section S1.3; the primary area-based metrics (Section 4.6.4) are computed directly from the discrete per-ROI classifications and are therefore independent of k.

S1.7. Inference Parameter Adjustments for the IM15 Evaluation

Because IM15 was acquired at a slightly lower spatial resolution (0.3 µm/pixel vs. 0.227 µm/pixel), the resolution-dependent parameters of the inference pipeline were adjusted accordingly. The local and context crop sizes were rescaled from 256 × 256 and 512 × 512 to 192 × 192 and 384 × 384 pixels, preserving the physical field of view captured by each crop (Section 4.6.2). The neighbourhood hyperparameter k was kept at its default value of 12, yielding σ = 94 px for this image, as derived from the formula in Supplementary Section S1.6 and the observed ROI density.

The three detection parameters were jointly adjusted to accommodate the weaker and more spatially fragmented CX43 signal observed on this section. First, thresh_value was lowered from 180 to 150 to preserve faintly stained regions in the binary mask. Second, kernel_open was reduced from 9 to 5 because small isolated detections are more likely to correspond to genuine low-intensity signal than to noise under weak staining. Third, kernel_dilate was increased from 5 to 21 to allow fragmented regions to reconnect into biologically coherent ROIs. All three adjustments respond to a single underlying feature of the evaluation image: weaker and more fragmented CX43 signal than in the development set. The decision threshold τ was kept at its default value of 0.5. The complete set of adjusted parameter values is reported in Table 8 of the main text.

S1.8. MARTA Parameters for the IM15 Evaluation

Image IM15 was processed with MARTA [18] using the input parameters listed in Supplementary Table S5. Parameters were selected by the domain expert after iterative

review of intermediate segmentation outputs. Parameters not listed were kept at their default values as defined in the original MARTA implementation [18].

Supplementary Table S5. Input parameters used to run MARTA [18] on image IM15. c1, c2, and c3 denote the image channels as labelled by MARTA (verify channel correspondence to the fluorescence markers used in this study).

**Category**	**Parameter**	**Value**	**Unit**
**General**	Spatial resolution	0.3	µm/pixel
	Equalize inputs	Yes	—
	Processing mode	Automatic	—
	Channel combination mode	addch	—
	Box padding (h)	28	pixels
	Threshold binarization	70	—
**Morphological transforms — c1**	Kernel noise size	1	pixels
	Kernel growth size	3	pixels
	Iterations for growth	3	—
**Morphological transforms — c2**	Kernel noise size	1	pixels
	Kernel growth size	7	pixels
	Iterations for growth	4	—
**Morphological transforms — c3**	Kernel noise size	1	pixels
	Kernel growth size	3	pixels
	Iterations for growth	5	—
**Cell filters — first filtering**	Minimum contour area	100	μm^2
	Minimum contour perimeter	40	µm
**Cell filters — second filtering**	Minimum cardiomyocyte length	20	µm
	Maximum cardiomyocyte length	200	µm
	Minimum cardiomyocyte width	5	µm
	Maximum cardiomyocyte width	40	µm
	Minimum aspect ratio (length/width)	1	—
**Output / image processing**	Gamma correction value	0.5	—
	Reduction factor for overlapping output	0	—

S2. Supplementary Results

S2.1. Final Retrained Model Performance

To complement the held-out test results reported in the main manuscript, the performance of the final retrained model is summarized in Supplementary Table S6. This table reports overall discrimination metrics for the training, validation, and test subsets, together with class-wise precision and recall for the lateralized and terminal classes.

Supplementary Table S6. Performance summary of the final retrained model across the training, validation, and test subsets. ROC-AUC and PR-AUC are reported at the subset level. Class-wise precision and recall are shown for the lateralized (Lat.) and terminal (Term.) classes using the default decision threshold of 0.5. The training, validation, and test subsets contained 2966, 68, and 105 ROIs, respectively.

**Subset**	**ROC-AUC**	**PR-AUC**	**Prec. Lat.**	**Recall Lat.**	**Prec. Term.**	**Recall Term.**
Train	0.977	0.956	0.902	0.885	0.930	0.941
Validation	0.802	0.592	0.500	0.500	0.820	0.820
Test	0.904	0.808	0.727	0.667	0.904	0.926

The final retraining dynamics are shown in Supplementary Figure S3. Across the three-stage optimization procedure, training loss decreased progressively, with the most pronounced drop during stage 3 when the full network was unfrozen. Validation AUC improved over training and reached its maximum around the selected checkpoint, whereas validation loss achieved its minimum at epoch 13. This checkpoint was therefore retained for downstream test evaluation.

Slide-level confusion matrices for the training and validation sets are shown in Supplementary Figure S4. Classifier performance was not homogeneous across slides: IM133 showed near-perfect separation, while IM1315 showed substantially lower performance (precision and recall of 0.500 for the lateralized class), consistent with the heterogeneity observed in the held-out test slides.

The distribution of predicted probabilities in the training and validation sets is shown in Supplementary Figure S5. In both subsets, correct predictions tended to occupy more extreme probability ranges, whereas false negatives and false positives accumulated closer to the decision threshold, consistent with the ROI-level pattern observed in the held-out test set.

Note: validation metrics from the final retraining run are not expected to exactly match those from the Optuna search, as each search trial was summarized according to the minimum validation loss during that trial. The final checkpoint retained for test evaluation is the one achieving the lowest validation loss in the final retraining run.

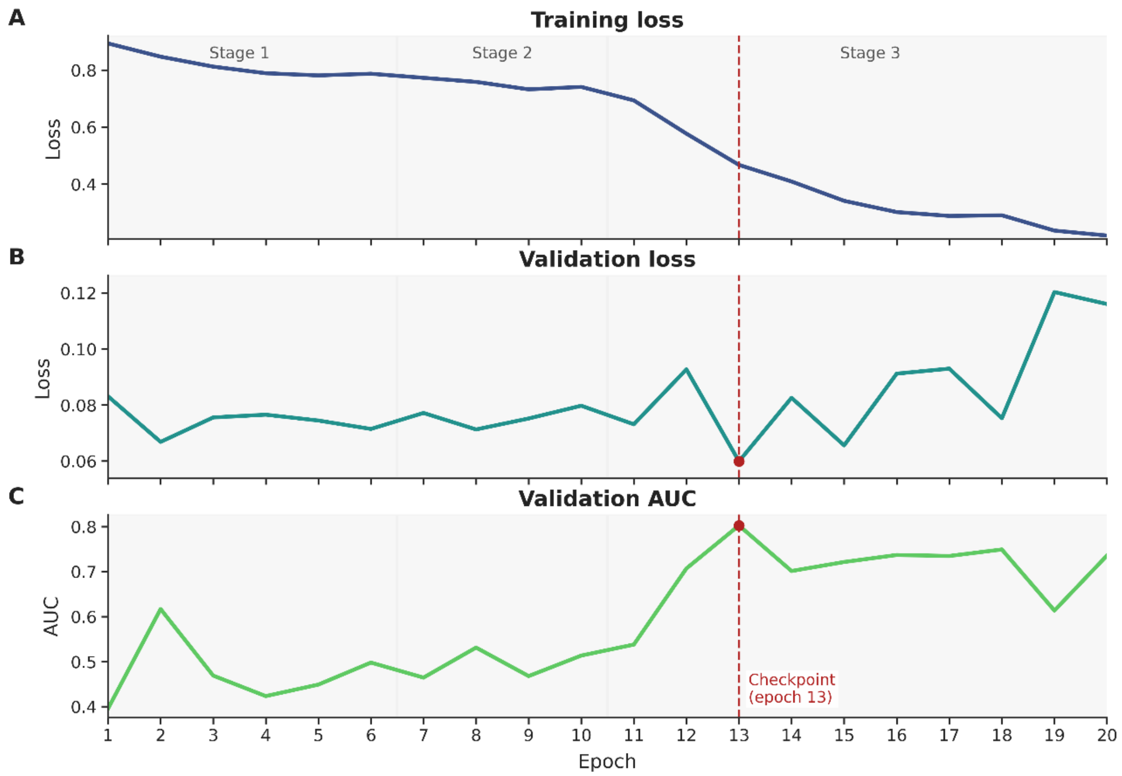

Supplementary Figure S3. Training dynamics of the final retrained model across the three-stage optimization procedure. (A) Training loss across epochs. (B) Validation loss across epochs. (C) Validation AUC across epochs. Background shading indicates the three training stages; the selected checkpoint is marked at the epoch with minimum validation loss.

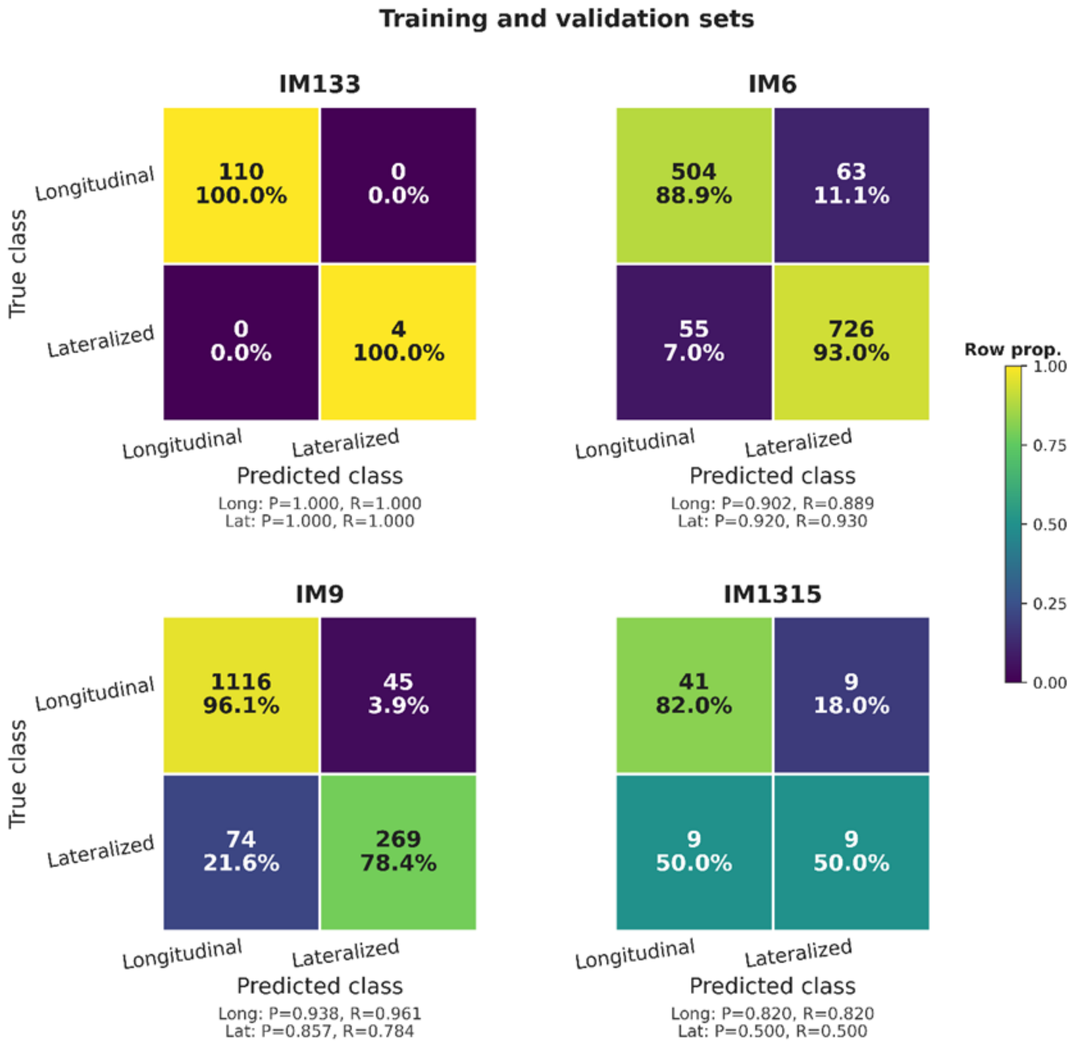

Supplementary Figure S4. Confusion matrices for the training and validation sets across slides IM133, IM6, IM9, and IM1315. Rows indicate true labels and columns indicate predicted labels. Cell colors show row-normalized proportions, allowing direct comparison across slides regardless of class imbalance. Numbers within each cell denote the absolute count and the corresponding within-row percentage. Precision and recall for the terminal and lateralized classes are reported below each panel.

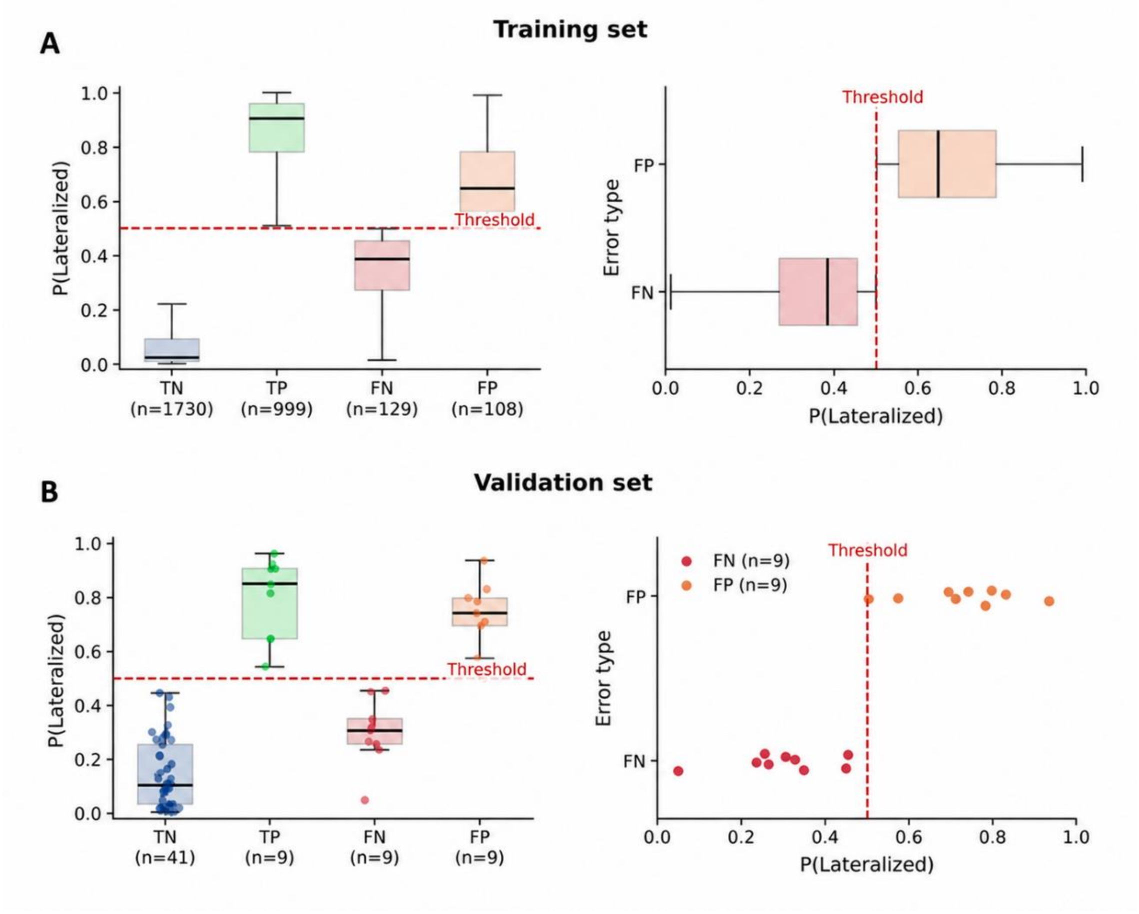

Supplementary Figure S5. Predicted probability distributions by outcome and error regime in the training and validation sets. (A) Training set. Left: distribution of P(lateralized) across true negatives (TN), true positives (TP), false negatives (FN), and false positives (FP). Right: predicted probabilities for misclassified samples only (FN and FP). (B) Validation set. Left: same layout with individual observations overlaid. Right: probabilities for misclassified validation samples. The dashed red line marks the decision threshold at 0.5 in all panels.

S2.2. Annotation Consistency Analysis

Overall annotation consistency was substantial for both the repeated annotation by the same expert and the independent annotation by a second expert (see Section 2.3 of the main manuscript). Slide-level agreement results are reported in Supplementary Table S7, and the corresponding confusion matrices are reported in Supplementary Table S8.

Agreement was not homogeneous across tissue sections. IM9 showed the lowest consistency in both analyses (intra-observer: 63.33%, κ = 0.267; inter-observer: 60.00%, κ = 0.200), suggesting that the sampled ROIs from IM9 were more morphologically ambiguous. By contrast, IM1313, IM1314, and IM1315 showed higher consistency, consistent with their lower prevalence of lateralized ROIs and their role as validation and test subsets in the main slide-level partitioning strategy. These per-slide values should be interpreted descriptively rather than as definitive reliability estimates, given the small number of ROIs per section (n = 30).

S2.2.1. Intra- and Inter-Observer Agreement

Supplementary Table S7. Slide-level annotation consistency results. (A) Intra-observer comparison (same expert, 12-month interval). (B) Inter-observer comparison (second expert). Overall intra-observer: percent agreement = 85.56%, Cohen's κ = 0.703. Overall inter-observer: percent agreement = 83.33%, Cohen's κ = 0.651.

A. Intra-observer comparison (same expert)

**Tissue section**	**n**	**Agreement (%)**	**Cohen's κ**
IM1313	30	93.33	0.760
IM1314	30	96.67	0.933
IM1315	30	86.67	0.733
IM133	30	83.33	0.453
IM6	30	90.00	0.800
IM9	30	63.33	0.267
Overall	180	85.56	0.703

B. Inter-observer comparison (second expert)

**Tissue section**	**n**	**Agreement (%)**	**Cohen's κ**
IM1313	30	93.33	0.760
IM1314	30	90.00	0.800
IM1315	30	76.67	0.533
IM133	30	93.33	0.712
IM6	30	86.67	0.733
IM9	30	60.00	0.200
Overall	180	83.33	0.651

Supplementary Table S8. Confusion matrices for the intra- and inter-observer annotation consistency comparisons (n = 180 ROIs each).

A. Intra-observer comparison (same expert)

**Original label**	**Repeat: terminal**	**Repeat: lateralized**
Terminal	93	18
Lateralized	8	61

B. Inter-observer comparison (second expert)

**Original label**	**Second expert: terminal**	**Second expert: lateralized**
Terminal	94	17
Lateralized	13	56

S2.2.2. Agreement Between Expert Reannotations Under the Same Interface

As an additional exploratory analysis, agreement between the two repeated annotation sets obtained with the interactive annotation tool was examined to characterize observer consistency under the same annotation interface. Agreement between the repeated annotations of the primary and secondary experts was 86.67%, with a Cohen's κ of 0.727, indicating substantial agreement. The corresponding confusion matrix is reported in Supplementary Table S9.

Although this comparison does not by itself establish the absence of interface-related bias, it supports the view that repeated expert annotation under the same standardized environment remained globally consistent.

Supplementary Table S9. Confusion matrix for the agreement between the primary and secondary expert annotations obtained using the interactive annotation tool (n = 180 ROIs; percent agreement = 86.67%, Cohen's κ = 0.727).

Primary expert	Second expert: terminal	Second expert: lateralized
Terminal	92	9
Lateralized	15	64

S2.3. Area-Based and Heatmap-Based Estimates of Global Percent Lateralization

Supplementary Table S10 reports the global lateralization metrics computed under the two aggregation schemes described in Section 4.6.4 of the main text for the three tissue sections analyzed with the inference pipeline. Both families of metrics use the CX43-positive mask as their common support but differ in how predictions are aggregated: $\%LatArea_{all}$ counts each ROI as fully lateralized or fully terminal once its predicted probability crosses the decision threshold $\tau = 0.5$, whereas $\%LatHeat_{all}$ averages the continuous probability field $H(x,y)$ pixel-wise over the same mask.

Supplementary Table S10. Comparison of area-based and heatmap-based global lateralization metrics across the three analyzed tissue sections. All sections were processed with the default neighbourhood parameter k = 12; the corresponding kernel standard deviation σ was derived independently for each image from its observed ROI density following Supplementary Section S1.6, yielding σ = 126 px for IM1313 and IM1314 and σ = 94 px for IM15. Diff: absolute difference in percentage points.

Section	$\%LatArea_{a\ell\ell}$	$\%LatHeat_{a\ell\ell}$	Diff (pp)
IM1313	6.63%	9.35%	+2.72
IM1314	19.06%	25.66%	+6.60
IM15	44.26%	49.74%	+5.48

Across all sections, $\%LatHeat_{a\ell\ell}$ was systematically higher than $\%LatArea_{a\ell\ell}$, with absolute differences ranging from 2.72 to 6.60 pp. This offset reflects the different aggregation operations underlying each metric: the Gaussian smoothing used to construct $H(x,y)$ diffuses probability mass from high-probability ROIs into their local neighbourhood, raising the pixel-wise mean relative to the hard-thresholded area-based estimate. The close correspondence across all three sections (differences ≤ 7 pp) indicates that both families of metrics capture the same underlying global lateralization burden, supporting the use of %LatArea as the primary reported summary with %LatHeat as a complementary confidence-weighted check.

S3. Supplementary Analysis of Representative ROI Crops

S3.1. Representative ROI-Level Examples Underlying the Spatial Error Analysis

Supplementary Figures S6 and S7 show representative 256- and 512-pixel ROI crops selected to illustrate clear, borderline, and morphologically challenging cases discussed in the main text. The identifiers, error types, and bounding-box coordinates for the displayed cases are listed in Supplementary Table S11.

Some examples correspond to clearly resolved predictions associated with extreme model probabilities. In IM1313, clear TN examples (2974, 2997, and 3002) show very low lateral probabilities; in IM1314, clear TN and TP examples are likewise associated with high model confidence. Together, these cases support the idea that the model behaves robustly when the morphological pattern is well defined.

Other examples are better interpreted as borderline: ROI 3012 in IM1313 and ROIs 3028 and 3034 in IM1314 appear difficult to classify with full confidence on visual inspection, suggesting continuity between clear and ambiguous cases. A further group of discordant ROIs is morphologically challenging: in IM1313, ROIs 3011, 3010, 3009, and 3013 include dim, partially out-of-focus, or locally complex patterns; in IM1314, challenging FN examples include 3052, 3055, 3063, and 3069. The FP pair 3018–3019 may represent the same local phenomenon, and some cases may reflect annotation uncertainty.

Supplementary Table S11. ROI identifiers and bounding-box coordinates for the zoomed examples shown in the spatial error analysis (Figure 7 of the main text).

Slide	Zoom	ROI ID	Error type	x_1	y_1	x_2	y_2
IM1313	Z1	3010	FN	8027	2701	8126	2804
IM1313	Z1	2982	TN	7984	2701	8085	2803
IM1313	Z1	2983	TN	7966	2681	8063	2778
IM1313	Z1	2984	TN	7916	2633	8053	2754
IM1313	Z1	2985	TN	8127	2852	8224	2950
IM1313	Z1	2986	TN	8179	2565	8291	2713
IM1314	Z1	3055	FN	8151	3294	8262	3412
IM1314	Z1	3017	TN	8098	3399	8226	3592
IM1314	Z1	3030	TN	8112	3660	8233	3825
IM1314	Z1	3054	TP	8195	3344	8294	3445
IM1314	Z1	3056	TP	8278	3485	8378	3585
IM1314	Z1	3057	TP	8247	3439	8356	3554
IM1314	Z2	3063	FN	8277	2054	8389	2175
IM1314	Z2	3064	TP	8190	2015	8288	2112
IM1314	Z2	3065	TP	8125	1986	8269	2099
IM1314	Z3	3028	FP	9431	1702	9546	1797
IM1314	Z3	3029	TN	9515	1691	9626	1797
IM1314	Z4	3069	FN	10958	4316	11057	4415
IM1314	Z4	3070	TP	10997	4248	11099	4350

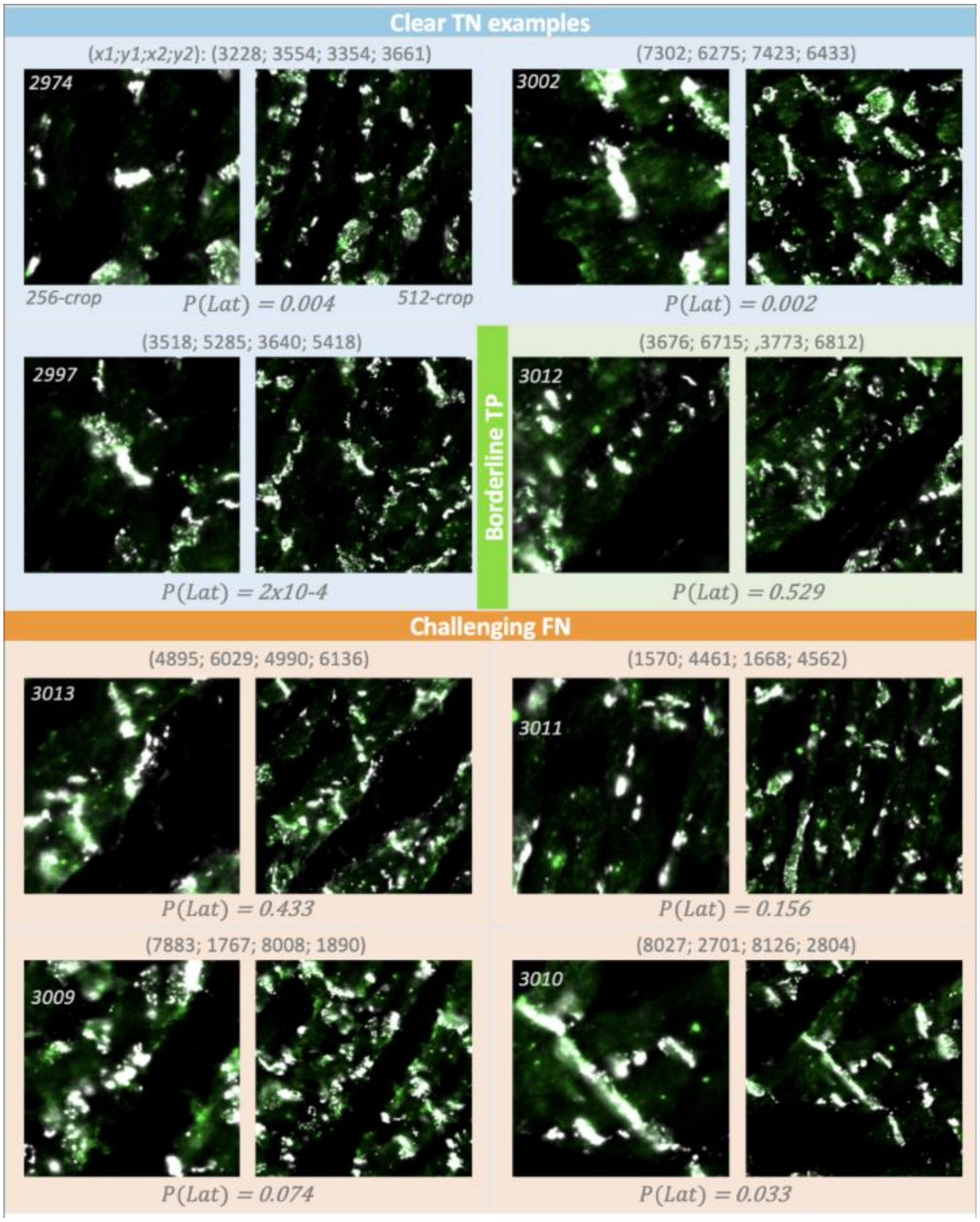

Supplementary Figure S6. Representative ROI crops from held-out test slide IM1313, including clear true negatives (TN), a borderline true positive (TP), and challenging false negatives (FN). For each ROI, a local 256 × 256 crop and a wider 512 × 512 contextual crop are shown side by side. Bounding-box coordinates on the original slide are indicated above each example as (x_1, y_1, x_2, y_2), the ROI identifier is shown in the upper-left corner, and the model-predicted probability P(Lat) is reported below.

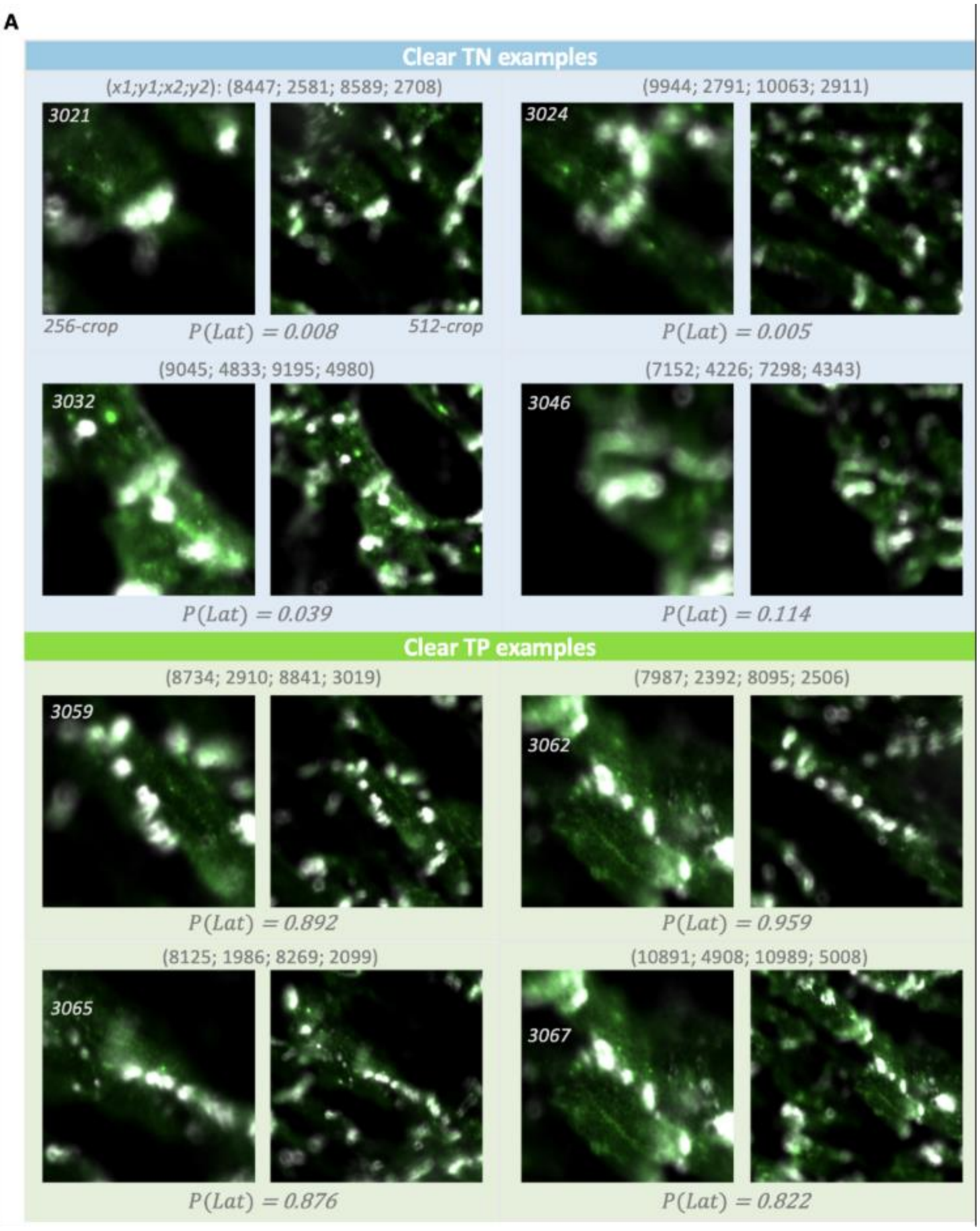
A
Clear TN examples
(x1;y1;x2;y2): (8447; 2581; 8589; 2708)
3021
256-crop
P(Lat) = 0.008
512-crop
(9944; 2791; 10063; 2911)
3024
P(Lat) = 0.005
(9045; 4833; 9195; 4980)
3032
P(Lat) = 0.039
(7152; 4226; 7298; 4343)
3046
P(Lat) = 0.114
Clear TP examples
(8734; 2910; 8841; 3019)
3059
P(Lat) = 0.892
(7987; 2392; 8095; 2506)
3062
P(Lat) = 0.959
(8125; 1986; 8269; 2099)
3065
P(Lat) = 0.876
(10891; 4908; 10989; 5008)
3067
P(Lat) = 0.822

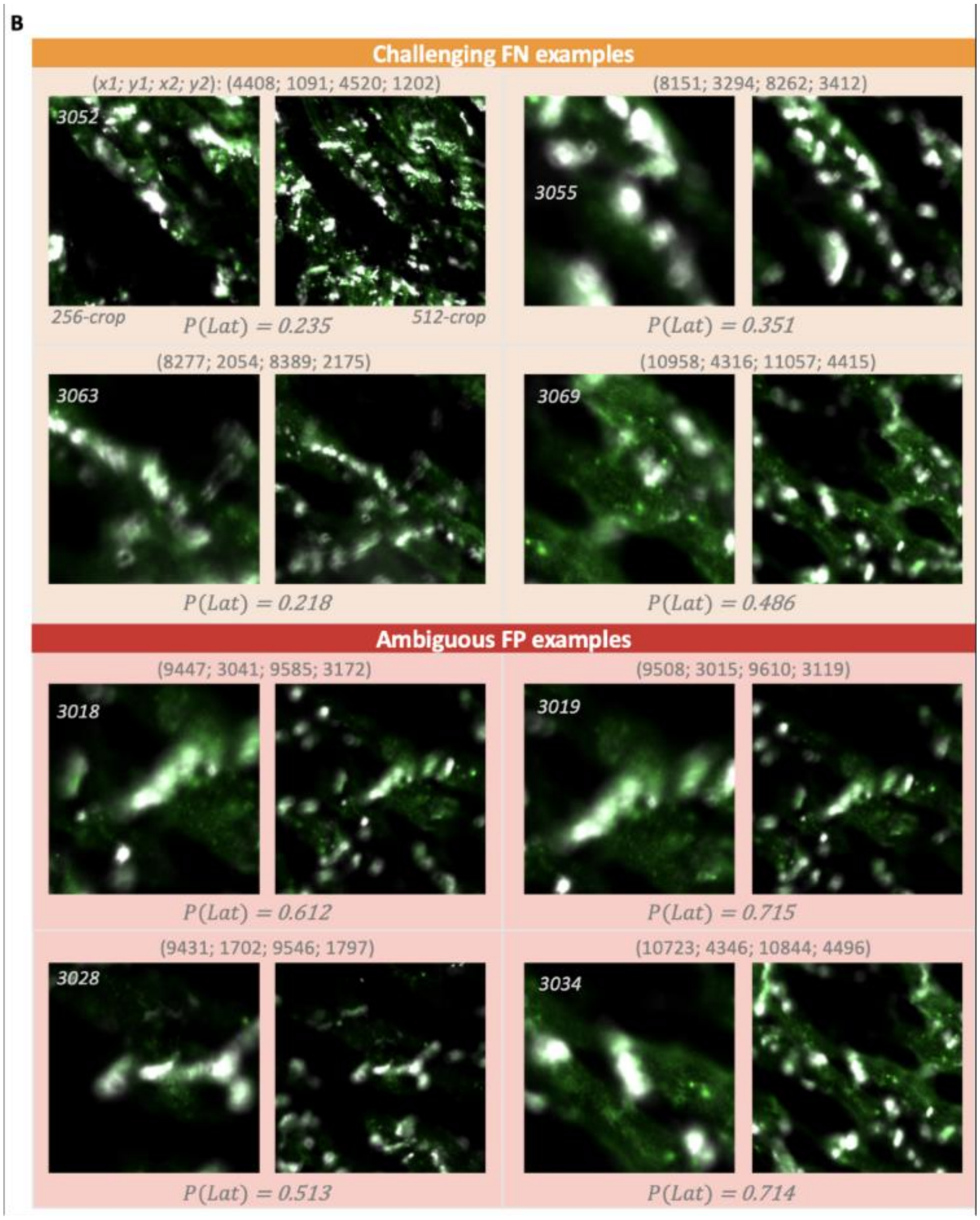

Supplementary Figure S7. Representative ROI crops from held-out test slide IM1314. (A) Clear true negatives (TN) and clear true positives (TP). (B) Challenging false negatives (FN) and ambiguous false positives (FP). Layout as in Supplementary Figure S6.

S4. Supplementary Analyses

S4.1. ROI-Level Partitioning Sensitivity Analysis

As a complementary sensitivity analysis, the annotated dataset was also partitioned at the ROI level using stratified random sampling. This analysis was not intended to replace the primary slide-level evaluation, but to assess whether the main model-selection trends remained qualitatively stable under a less stringent partitioning scheme.

S4.1.1. Alternative ROI-Level Partitioning Strategy

The dataset was partitioned using a two-step stratified random procedure: 15% of ROIs were reserved as a held-out test set; the remaining 85% were divided into training (85%) and validation (15%) subsets. Unlike slide-level partitioning, ROI-level splitting allows ROIs from the same tissue section to appear in different subsets, reducing independence between training and evaluation data and potentially yielding optimistically biased performance estimates. The resulting subset composition and per-slide ROI distribution are reported in Supplementary Table S12. Two Optuna studies were performed using the same fixed parameters and search spaces as in the main analysis (Supplementary Tables S1 and S2).

Supplementary Table S12. ROI-level partitioning: subset composition (A) and per-slide ROI distribution (B).

A. Subset composition

Subset	n (ROIs)	Terminal (%)	Lateralized (%)
Training	2267	62.73	37.27
Validation	401	62.84	37.16
Test	471	62.63	37.37

B. Per-slide ROI distribution across subsets

Section	Train	Val	Test	Total
IM6	997	171	180	1348
IM9	1063	192	249	1504
IM133	78	16	20	114
IM1313	33	8	7	48
IM1314	43	9	5	57
IM1315	53	5	10	68

S4.1.2. Sensitivity Analysis Results

S4.1.2.1. ROI-level architecture search

Under ROI-level partitioning, the main qualitative conclusion that contextual information is beneficial remained unchanged. However, the best-performing architecture was a contextual-only model with an MLP head (minimum validation loss = 0.0310, validation AUC = 0.9179), rather than the dual-scale model selected under slide-level partitioning. Full trial-wise results are in Supplementary File 2 (architecture). This shift suggests that the added value of explicitly combining local and contextual views depends on the partitioning strategy: under ROI-level splitting, section-specific contextual information may be easier to exploit, making the contextual-only formulation sufficient. The advantage of MLP heads over logistic heads was retained.

S4.1.2.2. Fine-tuning under ROI-level partitioning

After fixing the contextual-only architecture, fine-tuning further improved validation performance: minimum validation loss decreased by ≈11% to 0.0276, and validation AUC reached 0.9312. As in the primary analysis, the best checkpoint was in stage 3 and a

discriminative learning-rate hierarchy was favored. Full trial-wise results are in Supplementary File 2 (fine-tuning). As expected, absolute validation metrics were consistently higher than under slide-level partitioning, reflecting the less stringent partitioning scheme rather than improved real-world generalization. These results confirm that the usefulness of contextual information and progressive fine-tuning is robust, while further supporting slide-level partitioning as the primary evaluation framework.

S5. Data Availability

The source code used in this study is publicly available in the project GitHub repository. The pretrained model checkpoint is publicly available on Hugging Face and is intended to be used together with the main repository documentation and inference pipeline. The fluorescence images used in this study are publicly available through Zenodo. Additional implementation details, including instructions for reproducing the pipeline, applying the pretrained model, training from scratch on new data, and using the expert annotation tool, are provided in the public code repository.

GitHub repository: https://github.com/dgattari/CLARISA.git

Hugging Face model checkpoint: https://huggingface.co/jsanchoz/marta-cx43-slide-classifier

Zenodo record (fluorescence images): https://doi.org/10.5281/zenodo.19664101

S6. Computational Resources

All model training experiments were conducted on a system equipped with 64 CPUs and one NVIDIA A100 80 GB PCIe GPU. The total tracked compute time for the final model training run was 14 min 55 s.
